# Predicting Rift Valley Fever Inter-epidemic Activities and Outbreak Patterns: Insights from a Stochastic Host-Vector Model

**DOI:** 10.1371/journal.pntd.0005167

**Published:** 2016-12-21

**Authors:** Sansao A. Pedro, Shirley Abelman, Henri E. Z. Tonnang

**Affiliations:** 1 School of Computer Science and Applied Mathematics, University of the Witwatersrand, Johannesburg, South Africa; 2 Modelling Unit, International Center of Insect Physiology and Ecology, Nairobi, Kenya; 3 Departmento de Matemática e Informática, Universidade Eduardo Mondlane, Maputo, Mozambique; 4 International Maize and Wheat Improvement Center (CIMMYT) ICRAF House, United Nation, Avenue, Gigiri, Nairobi, Kenya; Colorado State University, UNITED STATES

## Abstract

Rift Valley fever (RVF) outbreaks are recurrent, occurring at irregular intervals of up to 15 years at least in East Africa. Between outbreaks disease inter-epidemic activities exist and occur at low levels and are maintained by female *Aedes mcintoshi* mosquitoes which transmit the virus to their eggs leading to disease persistence during unfavourable seasons. Here we formulate and analyse a full stochastic host-vector model with two routes of transmission: vertical and horizontal. By applying branching process theory we establish novel relationships between the basic reproduction number, *R*_0_, vertical transmission and the invasion and extinction probabilities. Optimum climatic conditions and presence of mosquitoes have not fully explained the irregular oscillatory behaviour of RVF outbreaks. Using our model without seasonality and applying van Kampen system-size expansion techniques, we provide an analytical expression for the spectrum of stochastic fluctuations, revealing how outbreaks multi-year periodicity varies with the vertical transmission. Our theory predicts complex fluctuations with a dominant period of 1 to 10 years which essentially depends on the efficiency of vertical transmission. Our predictions are then compared to temporal patterns of disease outbreaks in Tanzania, Kenya and South Africa. Our analyses show that interaction between nonlinearity, stochasticity and vertical transmission provides a simple but plausible explanation for the irregular oscillatory nature of RVF outbreaks. Therefore, we argue that while rainfall might be the major determinant for the onset and switch-off of an outbreak, the occurrence of a particular outbreak is also a result of a build up phenomena that is correlated to vertical transmission efficiency.

## Author Summary

Rift Valley fever (RVF) is a relatively novel vector-borne zoonotic disease, with long and irregular periods between outbreaks. Although outbreaks are highly correlated with occurrence of abnormal rainfall and flooding regimes, there have been instances with above normal rainfall where no disease outbreaks were recorded. This suggests that while rainfall might be the major determinant for the onset and switch-off of an outbreak, it may not be the only factor responsible for the temporal characteristic pattern of RVF outbreaks. We suspect that apart from influence of environmental conditions, disease outbreaks result from build up phenomena that depend on vertical transmission efficiency. Therefore, in this study we focus on formulating a stochastic host-vector model with two routes of transmission (horizontal and vertical) without seasonality to investigate the role of vertical transmission in the dynamics of the disease. First we apply branching process theory to characterize the relationships between vertical transmission and invasion probability and between vertical transmission and extinction probability. Finally, we apply an analytic approach, van Kampen’s system size expansion method to characterize disease complex fluctuations that are simply a result of interaction between demographic stochasticity, non-linearity and vertical transmission efficiency. Our results show that it is possible to reduce the frequency and intensity of RVF outbreaks by simply reducing the efficiency of vertical transmission.

## Introduction

Rift Valley fever (RVF) is an emerging zoonotic disease with pronounced health and economic impacts, particularly to vulnerable African communities with low resilience to economic and environmental challenges [1–3]. Studies have shown that the disease has two distinct cycles: the epizootic/epidemic and the enzootic/inter-epidemic or endemic [4]. During the inter-epidemic cycle, disease transmission occurs at low levels in nature during periods of average rainfall. The virus is thought to be maintained through transovarial transmission from the female *Aedes* mosquito to her eggs and by occasional amplification cycles in nearby livestock [5]. The epidemic activities have been found to be highly correlated to heavy rainfall and flooding (in particular in eastern and southern regions of Africa) that stimulate hatching of *Aedes* mosquito eggs, resulting in a massive emergence of both uninfected and infected *Aedes* mosquitoes [4, 6]. The infected ones if feeding on nearby vulnerable ruminants/livestock, would then trigger virus amplification, leading to an epizootic. An epizootic is mainly driven by the subsequent elevation of various *Culex* mosquito populations, which serve as excellent secondary vectors if immature mosquito habitats remain flooded for a long enough period [6, 7]. These disease epidemic activities occur at very irregular intervals of up to 15 years in the southern and eastern regions of Africa as well as in the horn of Africa [1, 3].

This characteristic temporal pattern of disease outbreaks adds an additional complication towards efforts for understanding and predicting occurrence of outbreaks. Findings from a pioneering empirical study in Tanzania on the subject of disease temporal and spatial patterns [7] suggest that continuous endemicity of Rift Valley fever virus (RVFV) may lead to periodic disease outbreaks. Similar observations have also been reported in Kenya [3] and South Africa [8, 9]. Although correlation between RVF outbreaks and the warm phase of El Niño/Southern Oscillation (ENSO) phenomena which lead to abnormal rainfall has been reported [10], there have been instances where no outbreaks were recorded following seasons of exceptionally above normal rainfall [7]. Moreover, in some Sub-Saharan regions, such as West Africa RVF outbreaks are not known to be correlated with above average rainfall [2]. In Senegal, it is reported that disease outbreaks have occurred during drought and normal rainy seasons [11, 12], and have been attributed to human-induced movement of livestock and trade and loss of herd immunity over time [11, 13]. However, a common situation could be the mechanism that lead to virus endemicity during dry season which is also suspected to be through transovarian transmission in *Aedes vexans* female mosquitoes [11]. The inter-epidemic period in Senegal is estimated to be 5-7 years, a time length thought to closely correspond to the time it takes for renewal of a domestic herd of ruminants [12]. This suggests that while rainfall might be the major determinant factor for the onset and switch-off of an outbreak, it is likely to not be the only factor responsible for this temporal characteristic pattern of disease outbreaks. Although consensus is yet to be achieved, studies have suggested that causal association between local environment factors, livestock density and movement, and encroachment of mosquitoes into new geographical area might be responsible for modifying temporal patterns of RVF outbreaks [14–16]. Findings by recent studies [7, 17] suggested that once RVFV had been introduced to a new geographical area, it becomes endemic and also pointed out that these newly established endemic areas constitute a source for future outbreaks once favourable environmental conditions are satisfied. Clearly, here the role of transovarial transmission cannot be neglected because it is essential for infection reactivation and scale of virus transmission in response to climatic conditions. This leads to a suspicion that apart from environmental conditions and other factors disease outbreak may be a result of a build up phenomena that depend on the efficiency of vertical transmission. Therefore, the present research study aims to investigate factors underlying the characteristic temporal patterns of RVF outbreaks and explore possibilities of predicting these outbreak patterns based on disease inter-epidemic activities.

Over the past decades mathematical models have been used to translate assumptions concerning transmission and spread of RVF at population level. From the pioneer RVF models by Favier et al. [18] and Gaff et al. [19], several models have been formulated and analysed using deterministic compartmental modelling approach [20–28]. Although these models have potential for examining factors underlying dynamics of the disease, they fail to capture observed fluctuations on the occurrence of RVF outbreaks. Nevertheless, extending these models to include seasonality yielded rich dynamics including chaotic behaviour [28]. Chitnis et al. [24] suggested that seasonality combined with mosquito vertical transmission and/or introduction of new infected individuals after immunity wanes was necessary for the survival of RVF and inter-epidemic persistence. On the other hand a study in [29] used a seasonally forced deterministic model to explore different scenarios of infection persistence including vertical transmission and alternate wildlife hosts, and concluded that RVF persistence is a delicate balance between numerous species of susceptible hosts, mosquito species, vertical transmission and environmental stochasticity. In these situations such dynamics are attributed to climatic variations disregarding the fact that interaction between the deterministic dynamics and demographic stochasticity is central for explaining realistic disease patterns [30]. Deterministic models are typically assumed to be reasonable approximations for infinitely large homogeneous populations, and arise from the analysis of mean field stochastic models, such that if one considers finite populations which is the case of livestock, stochastic interactions even within a well-mixed system may introduce new phenomena [31]. Therefore, it is more likely that these disease characteristic temporal patterns could be captured by fully stochastic models [31, 32], which are known to show large oscillations caused by the stochasticity exciting the system’s natural frequency [33, 34]. Stochastic effects are known to show major impacts whenever the prevalence of infection in either the host or vector population, or both are low and can be highly significant during the period immediately after the introduction of infection into a population [35].

In this study we formulate a full host-vector stochastic model which takes into account mechanisms of vertical transmission on the vector population. Our aim is to examine the impact of stochastic effects and virus endemicity on the invasion and persistence of the disease. Stochastic effects can also lead to disease extinction during endemic settings [36]. To investigate these situations we employ branching process theory [37–39], which has been successfully applied in vector-borne epidemic models (for more details see [35, 40]). Here we extend the analysis presented in [35] to include vertical transmission while implementing infection rates that depend on the sizes of both host and vector populations. Our objective is to examine the impacts of mosquito biting behaviour and host efforts to avoid the biting on the invasion and persistence of the disease in the presence of vertical transmission. Although stochasticity can cause large departures from equilibrium, potentially allowing the number of infectives to fall to low levels [35], it could act passively to kick the system between different deterministic states [41], as well as interacting with the non-linearity to excite the transients [32], leading to either periodic or non-periodic oscillations. Using power spectra analysis we investigate the periodicity of fluctuations of RVF outbreaks as was undertaken for avian influenza in [31]. This is accomplished by formulating the model as a master equation which is then studied using van Kampen’s system size expansion [42], to provide a prediction for the dominant period of disease oscillations. Since the macroscopic dynamics can then be viewed as a sum of a deterministic and a stochastic part, this approach provides a unique opportunity to investigate the effects of stochasticity on disease endemicity and outbreaks. The approach has been successfully applied while investigating the effects of stochastic amplification [34, 43] and seasonal forcing [32, 44, 45] on disease outbreaks in particular in childhood diseases and more recently on avian influenza [31]. Our objective here is to test ideas about whether the oscillatory patterns of disease outbreaks can be predicted by simply looking at disease inter-epidemic activities. Based on historical data of occurrence of disease outbreaks in particular in Kenya, Tanzania and South Africa, we suspect vertical transmission and chance events to influence the observed characteristic pattern of disease outbreaks. This analysis provides prediction of the dominant period of disease fluctuations depending on the efficiency of vertical transmission. The results highlight the role of continuous RVFV endemicity driven by vertical transmission on mosquitoes, on the periodicity of disease outbreaks which agree with findings from empirical studies [3, 7, 9]. Therefore, it is reasonable to argue that it could be possible to reduce the frequency and intensity of RVF outbreaks by controlling transovarial transmission efficiency.

## Methods

### RVF stochastic host-vector model with vertical transmission

To analytically investigate temporal dynamics of a RVF model by means of stochastic processes we formulate a simple but realistic stochastic host-vector model that captures all important features of RVF dynamics. The present study does not use primary data (medical records or public records), rather during model development we calibrate the model towards temporal characteristic patterns of RVF epidemic and inter-epidemic activities observed in East Africa and Southern Africa. In particular, the data used reflect patterns observed in Kenya, Tanzania and South Africa (see [3, 7, 9, 46, 47] and references therein). A description of all model parameters and their respective values, ranges and sources is given in Table 1.

**Table 1:** The parameters for the RVF model for high rainfall and moderate temperature (wet season) for model in Table 2 with values, range and references. Note that all parameter units are days. The parameter *α*_1_ is a function of the mosquito’s gonotrophic cycle (the amount of time a mosquito requires to produce eggs) and its preference for livestock blood, while *α*_2_ is a function of the ruminant’s exposed surface area, the efforts it takes to prevent mosquito bites (such as swishing its tail), and any vector control interventions in place to kill mosquitoes encountering cows or prevent bites [24].

Parameter	Description	Units	Baseline	Range	Reference
1/*μ*_1_	Mosquito life span	Days	20	10-30	[24, 50]
1/*μ*_2_	Livestock life span	Days	2190	360-3600	[19]
*q*_1_	Probability of vertical transmission		0.1	0-1	[51]
*α*_1_	Number of times a mosquito would like to bite a host	Days^−1^	0.33	0.1-0.5	[24, 52]
*α*_2_	Number of bites a host can sustain	Days^−1^	19	0.1-50	[24]
*α*	Biting rate	Days^−1^	0.71	0.1-0.8	[53]
*β*_21_	Probability of successful infection in livestock		0.21	0.001-0.54	[2, 24, 49]
*β*_12_	Probability of successful infection in mosquitoes		0.51	0.3-0.9	[2, 24, 49]
1/*ϵ*_2_	Infectious duration in livestock	Days	4	1-7	[24, 52, 54, 55]
*m*_0_	The ratio female mosquitoes to hosts		1.5	0-5	[56]

We investigate both disease epidemic and inter-epidemic activities in a livestock population where the transmission of the infection is intermediated by *Aedes* mosquitoes only. Thus, neglecting the presence of *Culex* species which are known to be the secondary vectors of the disease as in [24]. *Aedes* mosquitoes are responsible for both initial spread and persistence of the disease since the female can transmit the virus transovarially to her eggs [2, 48]. The mosquito sub-model is an *SI* type model, that is, with only two compartments: susceptible and infectious. This way we ignore the exposed class and mosquitoes once infected remain infected for life. The livestock sub-model is an *SIR* type model, that is, susceptible, infectious and recovered.

**Fig 1:**
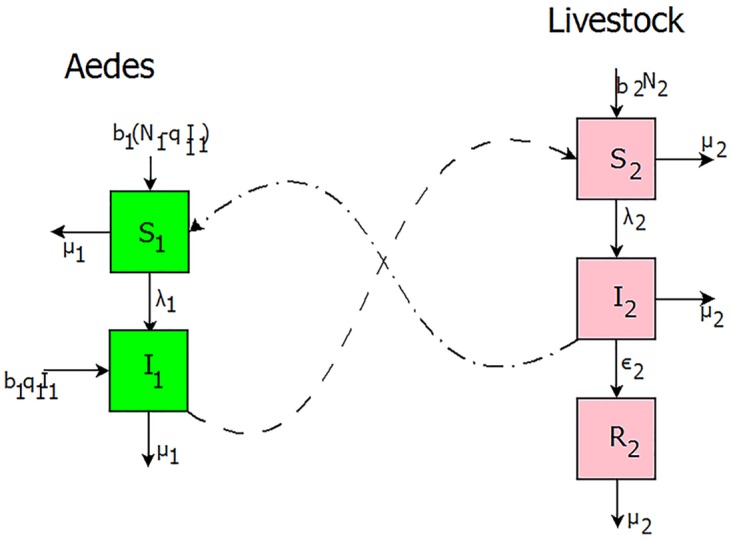
Flow diagram of RVF model with both vertical and horizontal transmission. Susceptible livestock, *S*_2_, acquire infection and move to compartment *I*_2_ when they are bitten by an *Aedes* infectious mosquito *I*_1_. They then recover with a constant per capita recovery rate to enter the recovered compartment, *R*_2_, class. Susceptible mosquito vectors, *S*_1_, become infected when they bite infectious livestock and progress to class *I*_1_. The solid lines represent the transition between compartments and the dashed lines represent the transmission between different species.

Animal hosts enter the susceptible class through birth at a constant rate, *μ*_2_. When an infectious *Aedes* mosquito bites a susceptible animal, there is a finite probability, *β*_21_ that the animal becomes infected. Once an animal host is successfully infected by an infected vector, it moves from susceptible class *S*_2_ to infectious class *I*_2_. After some time, the infectious animal host either recovers at rate *ϵ*_2_ and moves to recovered class, *R*_2_ or dies naturally at per capita rate of *μ*_2_. Female *Aedes* mosquitoes (we do not include male mosquitoes in our model because only female mosquitoes bite animals for blood meals) enter the susceptible class through birth at rate, *b*_1_. The term birth for mosquitoes accounts for and is proportional to the egg-laying rate; and survival of larvae [24]. Since most density-dependent survival of mosquitoes occurs in the larvae stage, we assume a constant emergence rate that is not affected by the number of eggs laid; that is, all emergence of new adult mosquitoes is limited by the availability of breeding sites [24]. Susceptible vectors, *S*_1_ are infected when they bite an infected animal with probability *β*_12_ and depending on the ambient temperature and humidity [49] the mosquitoes move from *S*_1_ to the infectious class, *I*_1_. To reflect the vertical transmission in *Aedes* mosquitoes a proportion of infected, *q*_1_ newly hatched mosquitoes joins class *I*_1_. Mosquitoes leave the population through a per capita natural death rate, *μ*_1_. Although births and deaths are intrinsically distinct events, we assume, for simplicity, that the vector birth and death rates have the same values, which means that the total population size *N*_1_ = *S*_1_ + *I*_1_ is kept constant. A key feature of the model is that the rate at which new infections occur in both host and vector is proportional to both host and vector population. That is, the total number of bites varies with both the host and vector population sizes. This allows more realistic modelling of situations where there is a high ratio of mosquitoes to livestock and where livestock availability to mosquitoes is reduced through control intervention as well as the efforts a host takes to prevent mosquito bites (such as swishing its tail) [24, 28]. Thus, the force of new infections in livestock is $\lambda_{21}=\frac{\alpha_{1}\alpha_{2}\beta_{21}I_{1}}{\alpha_{1}N_{1}+\alpha_{2}N_{2}}$ and the force of new infections in mosquitoes is $\lambda_{12}=\frac{\alpha_{1}\alpha_{2}\beta_{12}I_{2}}{\alpha_{1}N_{1}+\alpha_{2}N_{2}}$, where *α*_1_ is the number of times one *Aedes* mosquito would want to bite a host per day, if livestock were freely available (for details on their derivation see supplementary material section A). This is a function of the mosquitoes gonotrophic cycle (the amount of time a mosquito requires to produce eggs) and its preference for livestock blood. *α*_2_ is the maximum number of mosquito bites a host can sustain per day. This is a function of the hosts exposed surface area, the efforts it takes to prevent mosquito bites (such as swishing its tail), and any vector control interventions in place to kill mosquitoes encountering hosts or preventing bites [24]. This formalism allow us to evaluate how mosquito biting behaviour and vertical transmission in *Aedes* female mosquitoes impact both the probabilities of disease invasion and extinction and disease fluctuations. The former is accomplished by employing branching process theory which is central for determining critical epidemic behavioural thresholds [35], and for the later we used system-size expansion technique [57] and Fourier analysis. However, a standard incidence function used in mosquito transmitted diseases usually assumes that mosquitoes bite a particular host at a constant rate irrespective of the number of available hosts. Therefore, for very large *N*_2_ the above forces of infection can be approximated by the following standard incidence functions $\lambda_{21}^{\prime }=\beta_{21}\alpha m_{0}\frac{I_{1}}{N_{1}}$ and $\lambda_{12}^{\prime }=\beta_{12}\alpha \frac{I_{2}}{N_{2}}$ as the model forces of infections. In this case *α* is the mosquito biting rate, such that *α*/*N*_2_ is the rate at which a particular host is bitten by a particular mosquito, *m*_0_ = *N*_1_/*N*_2_ is the ratio female mosquitoes to hosts and *β*_21_ and *β*_12_ are the probabilities of successful transmission per bite [58, 59]. All the transitions of the host and the vector associated with their corresponding rates are illustrated graphically in Fig 1.

Setting the livestock population size to remain constant, we can omit the equation containing *R*_2_, since it can be obtained when *S*_2_ and *I*_2_ are known. Therefore, the basic ingredients of our new model framework are susceptible livestock *S*_2_, infected livestock *I*_2_ and infected *Aedes* mosquitoes *I*_1_. Unlike in deterministic models the numbers in these classes are no longer treated as continuous varying quantities [35], but instead as integers since individual-based stochastic models consider movements of individuals between classes to be discrete [60]. To be precise, these transitions are assumed to take place in a small time interval (*t*, *t* + Δ*t*) with inflows and outflows of magnitude unity. If we denote the numbers in each class as *s*_2_, *i*_2_ and *i*_1_ respectively, the general state of the system is then written as *σ* = (*s*_2_, *i*_2_, *i*_1_). Thus, *T*(*σ*′|*σ*) represents the transition probability per unit time from state *σ* to the state *σ*′. Note that we characterize the events taking place in the system into three distinct groups:

1. Infection
  $$\begin{array}{ccc} T(s_{2}-1,i_{2}+1,i_{1}|s_{2},i_{2},i_{1}) & = & \beta_{21}\alpha^{\prime }m_{0}\frac{i_{1}}{N_{1}}s_{2},\\ T(s_{2},i_{2},i_{1}+1|s_{2},i_{2},i_{1}) & = & \beta_{12}\alpha^{\prime }\frac{i_{2}}{N_{2}}\left(N_{1}-i_{1}\right), \end{array}$$ (1)
2. Birth/Death
  $$\begin{array}{ccc} T(s_{2}+1,i_{2},i_{1}|s_{2},i_{2},i_{1}) & = & \mu_{2}N_{2},\\ T(s_{2}-1,i_{2},i_{1}|s_{2},i_{2},i_{1}) & = & \mu_{2}s_{2},\\ T(s_{2},i_{2},i_{1}-1|s_{2},i_{2},i_{1}) & = & \mu_{1}i_{1}. \end{array}$$ (2)
3. Recovery
  $$\begin{array}{c} T\left(s_{2},i_{2}-1,i_{1}|s_{2},i_{2},i_{1}\right)=\left(\epsilon_{2}+\mu_{2}\right)i_{2}. \end{array}$$ (3)
  where $\alpha^{\prime }=\frac{\alpha_{1}\alpha_{2}}{\alpha_{1}m_{0}+\alpha_{2}}$ for general forces of infections *λ*_21_ and *λ*_12_, and *α*′ = *α* for standard forces of infections $\lambda_{21}^{\prime }$ and $\lambda_{12}^{\prime }$.

For better illustration we summarize all of the processes taking place in the system and their corresponding rates and probabilities of occurrence in Table 2. Note that these rates are the conditional instantaneous stochastic rates of individuals entering or leaving each compartment at time *t* and also depend on the sizes of each compartment.

**Table 2:** Stochastic model for vector-host disease system. The parameter *m*_0_ = *N*_1_/*N*_2_ is the ratio mosquitoes to hosts, and $\alpha^{\prime }=\frac{\alpha_{1}\alpha_{2}}{\alpha_{1}m_{0}+\alpha_{2}}$ is for general forces of infections *λ*_21_ and *λ*_12_, and *α*′ = *α* is for standard forces of infections $\lambda_{21}^{\prime }$ and $\lambda_{12}^{\prime }$.

Event	Transition	Rates of occurrence	Probability in [*t*, *t* + *dt*]
Birth of uninfected *Aedes*	*S*_1_ → *S*_1_ + 1	*μ*_1_(*N*_1_ − *q*_1_*I*_1_)	*μ*_1_(*N*_1_ − *q*_1_*I*_1_)*dt*
Infected *Aedes birth*	*I*_1_ → *I*_1_ + 1	*b*_1_*q*_1_*I*_1_	*b*_1_*q*_1_*I*_1_*dt*
Infection of susceptible *Aedes* from infectious host	*S*_1_ → *S*_1_ − 1, *I*_1_ → *I*_1_ + 1	$\beta_{12}\alpha^{\prime }\frac{I_{2}}{N_{2}}S_{1}$	$\beta_{12}\alpha^{\prime }\frac{I_{2}}{N_{2}}S_{1}dt$
Death of susceptible *Aedes*	*S*_1_ → *S*_1_ − 1	*μ*_1_*S*_1_	*μ*_1_*S*_1_*dt*
Death of infectious *Aedes*	*I*_1_ → *I*_1_ − 1	*μ*_1_*I*_1_	*μ*_1_*I*_1_*dt*
Birth of susceptible host	*S*_2_ → *S*_2_ + 1	*μ*_2_*S*_2_	*μ*_2_*S*_2_*dt*
Infection of susceptible host from infectious *Aedes*	*S*_2_ → *S*_2_ − 1, *I*_2_ → *I*_2_ + 1	$\beta_{21}\alpha^{\prime }m_{0}\frac{I_{1}}{N_{1}}S_{2}$	$\beta_{21}\alpha^{\prime }m_{0}\frac{I_{1}}{N_{1}}S_{2}dt$
Infectious host recovery	*I*_2_ → *I*_2_ − 1, *R*_2_ → *R*_2_ + 1	*ϵ*_2_*I*_2_	*ϵ*_2_*I*_2_*dt*
Death of susceptible host	*S*_2_ → *S*_2_ − 1	*μ*_2_*S*_2_	*μ*_2_*S*_2_*dt*
Death of infectious host	*I*_2_ → *I*_2_ − 1	(*m*_2_ + *μ*_2_)*I*_2_	(*m*_2_ + *μ*_2_)*I*_2_*dt*
Death of recovered host	*R*_2_ → *R*_2_ − 1	*μ*_2_*R*_2_	*μ*_2_*R*_2_*dt*

Using the probabilities in Table 2, we can now construct the master equation in its general form [34, 42, 61], describing temporal evolution of the probability distribution of determining the system in state *σ* at time *t*.

$$\begin{array}{c} \frac{dP(\sigma ;t)}{dt}=\sum_{\sigma^{\prime }\neq \sigma }T\left(\sigma |\sigma^{\prime }\right)P\left(\sigma^{\prime };t\right)-\sum_{\sigma^{\prime }\neq \sigma }T\left(\sigma^{\prime }|\sigma \right)P\left(\sigma ;t\right), \end{array}$$ (4)

where *σ* = (*s*_2_, *i*_2_, *i*_1_) represents the state of the system, *P*(*σ*, *t*) is the probability of the system in the state *σ* at time *t*. This can also be referred to as the forward Fokker-Planck (or forward Kolmogorov) equation, which is a differential equation for the probability density function *P*(*σ*, *t*) of determining the system in *σ* at time *t* and it cannot be solved exactly. An alternative analytical approach can be the derivation of the moments of the distribution of the state *σ*. However, for the purpose of our study we analyse the master equation using van Kampen’s system-size expansion [42], see Section C.2 of S1 Methods. In the following sections we determine both the probabilities of a major outbreak and extinction after introduction of a single or few infectives into a population that is otherwise susceptible.

### Estimating the probability of a major outbreak

In any disease model, a question of fundamental interest is to determine conditions under which a disease if introduced into a community with no immunity will develop into a large outbreak, and if it does, conditions under which the disease may become endemic. For this purpose, a key threshold parameter called the basic reproduction number, *R*_0_ is derived and analysed usually in deterministic epidemic models. In this context it is defined as the average number of secondary cases produced by a single infected individual during his or her entire infectious period, in a population which is entirely susceptible. In this regard, it is soon clear that when *R*_0_ < 1 each infected individual will produce less than one infected case and the probable result is that the disease will die out. On the contrary, if *R*_0_ > 1 each individual will produce more than one case and eventually the infection will invade the population. However, in the stochastic models, invasion of an infection into a susceptible population is not guaranteed by having *R*_0_ > 1: stochastic extinction can occur during the period immediately following introduction, when there are few infective individuals [35]. Thus, rather than the major outbreak that would be expected based on the behaviour of the deterministic model, only a minor outbreak might occur. During this early stage after the introduction of the pathogen, little depletion of susceptibles will have occurred and so probabilities of major outbreaks can be derived using the linear model that arises by assuming that the populations are entirely susceptible [62–64]. Thus, in the resulting model, the number of infectives can be approximated through a multi-type linear birth-death process [62]. In a multi-type branching process, individuals in the population are categorised into a finite number of types and each individual behaves independently [35]. An individual of a given type can produce offspring of possibly all types and individuals of the same type have the same offspring distribution [65, 66]. In our model the disease is spread via two modes of infection transmission: vertical and horizontal. Thus, an infectious mosquito produces an infected animal, and a proportion *q*_1_ of infectious mosquitoes produce infectives of the same type while an infected animal produces an infected mosquito. Therefore, by assuming that secondary infections arise independently and at a constant rate over the infectious period of each infective, then the distribution of secondary infections follow geometric distributions [35], with means $R_{0}^{11},R_{0}^{21}\;\mathrm{and}\;R_{0}^{12}$ for mosquito-to-mosquito, mosquito-to-animal and animal-to-mosquito transmission respectively (for more details see subsection B.2 of S1 Methods).

In this settings, for horizontal transmission the probability generating functions (PGF) for the joint distribution of the dynamic variables when a single infected mosquito was introduced at time 0 can be obtained and it is given by

$$\begin{array}{c} G_{i}\left(s\right)=E\left[\prod_{j=1}^{2}s_{j}^{X_{ij}}\right]. \end{array}$$ (5)

For vertical transmission the PGF is simply $G_{1}^{2}$ [67]. Note that {*X*_*ij*_, *i*, *j* = 1,2} is the number of infectives of type *j* produced by an infective of type *i*. *G*(*s*) is the probability generating function of the distribution of secondary infections and Eq (5) can be solved to find the extinction probability if there is initially one infective individual present. Extinction in the linear model is most likely to occur early in the process, so this corresponds to the occurrence of minor outbreaks in the nonlinear model, whereas non-extinction in the linear model corresponds to a major outbreak in the nonlinear model [35]. Eq (5) can be expanded to obtain the following formula [35],

$$\begin{array}{c} G_{i}\left(s_{1},s_{2}\right)=\sum_{k_{1},k_{2}}s_{1}^{k_{1}}s_{2}^{k_{2}}P\left(X_{i1}=k_{1},X_{i2}=k_{2}\right)=\frac{1}{1+\sum_{j=1}^{2}R_{ji}\left(1-s_{j}\right)} \end{array}$$ (6)

where *i* is equal to 1 or 2. An infective animal only directly give rise to secondary infections in the vector population. Thus, we have that *P*(*X*_21_ = *j*, *X*_22_ = *k*) is equal to *P*(*X*_21_ = *j*) when *k* = 0 and zero otherwise. Consequently the generating function *G*_2_(*s*_1_, *s*_2_) is a function of *s*_1_ alone,

$$\begin{array}{c} G_{2}\left(s_{1},s_{2}\right)=\frac{1}{1+R_{12}\left(1-s_{1}\right)}. \end{array}$$ (7)

However, when effects of vertical transmission are included, infective mosquitoes not only give rise to secondary infections in the animal population but also to secondary infection in the mosquito population through transmission from mother to eggs. Therefore, the generating function *G*_1_(*s*_1_, *s*_2_) is a function of *s*_1_ and *s*_2_,

$$\begin{array}{c} G_{1}\left(s_{1},s_{2}\right)=\frac{1}{1+R_{11}\left(1-s_{1}\right)+R_{21}\left(1-s_{2}\right)}. \end{array}$$ (8)

Extinction probabilities can be calculated by solving the pair of equations,

$$\begin{array}{c} G_{1}\left(G_{2}\left(s_{1},s_{2}\right)\right)=s_{1}\;\mathrm{and}\;G_{2}\left(G_{1}\left(s_{1},s_{2}\right)\right)=s_{2}, \end{array}$$ (9)

resulting from composition of functions in Eqs (7) and (8). The pair (*s*_1_, *s*_2_) = (1, 1) is always a solution. If *R*_0_ ≤ 1 it is the only solution, whereas for *R*_0_ > 1 there is another solution with both *s*_1_ and *s*_2_ being less than unity [38], where $R_{0}=\frac{q_{1}}{2}+\frac{1}{2}\sqrt{q_{1}^{2}+4R_{12}R_{21}}$ with $R_{12}=\frac{1}{\epsilon_{2}+m_{2}+\mu_{2}}\frac{\alpha_{1}\alpha_{2}\beta_{12}}{\alpha_{1}N_{1}+\alpha_{2}N_{2}}S_{1}^{0}$ being the number of new infections in *Aedes* mosquitoes generated by single infected animal and $R_{21}=\frac{\alpha_{1}\alpha_{2}\beta_{21}}{\alpha_{1}N_{1}+\alpha_{2}N_{2}}S_{2}^{0}\frac{1}{\mu_{1}}$ the number of new infections in animals generated by single infected *Aedes* mosquito.

### System size expansion of the stochastic host-vector model

So far we have formulated a fully stochastic host-vector model with both horizontal and vertical transmission, under well-mixed conditions and constructed the master Eq (4). To analyse the model we apply two methods: one is to simulate the system using the Gillespie algorithm [68], which gives the exact realization of temporal disease evolution. The other is analytical and consists of performing van Kampen’s system-size expansion [34, 42] of the master equation, which allows for quantitative prediction of the power spectrum of the time fluctuations of each of the system variables, and, therefore, of the dominant period of disease outbreaks [31]. Full details of van Kampen’s system size expansion are discussed in Section C of S1 Methods. This method allows us to derive analytical approximate solutions which involves making the following substitutions,

$$\begin{array}{ccc} s_{2} & = & N_{2}\phi_{1}+\sqrt{N_{2}}x_{1},\\ i_{2} & = & N_{2}\phi_{2}+\sqrt{N_{2}}x_{2},\\ i_{1} & = & N_{1}\psi +\sqrt{N_{1}}x_{3}, \end{array}$$

where *ϕ*_1_, *ϕ*_2_, *ψ* are fractions of the susceptible livestock, the infected livestock and infected *Aedes* mosquitoes respectively, with *x*_*l*_(*l* = 1, 2, 3) describing the stochastic corrections to the variables *s*_2_, *i*_2_, *i*_1_. This expands the master equation in powers of $N_{1}^{-1/2}$ and $N_{2}^{-1/2}$, such that the probability distribution *P*(*s*_2_, *i*_2_, *i*_1_;*t*) can be written in terms of the new variables *x*_1_, *x*_2_, *x*_3_. Then, in comparison to the leading order, yield the following deterministic system in terms of fractions as follows:

$$\begin{array}{ccc} \frac{d\phi_{1}}{dt} & = & -\beta_{21}\alpha^{\prime }m_{0}\psi \phi_{1}+\mu_{2}\left(1-\phi_{1}\right),\\ \frac{d\phi_{2}}{dt} & = & \beta_{21}\alpha^{\prime }m_{0}\psi \phi_{1}-\left(\epsilon_{2}+\mu_{2}\right)\phi_{2},\\ \frac{d\psi }{dt} & = & \beta_{12}\alpha^{\prime }\phi_{2}\left(1-\psi \right)+\mu_{1}q_{1}\psi -\mu_{1}\psi . \end{array}$$ (10)

**Fig 2:**
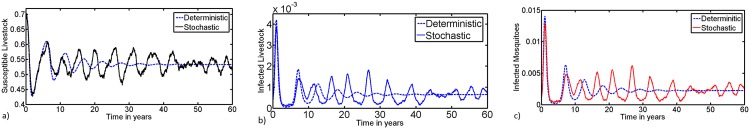
Realization of the RVF host-vector stochastic model and its deterministic counterpart. The trajectories of the deterministic counterpart are generated by integrating the mean field Eq (10). The values of the parameters in years are as follows: *q*_1_ = 0.2, *μ*_1_ = (1/20) ∗ 360, *μ*_2_ = 1/8, *β*_12_ = 0.194, *β*_21_ = 0.128, *ϵ*_2_ = (1/4) ∗ 360, *α*′ = *α* = 256, *m*_0_ = 1.5 and *R*_0_ = 1.8809, and their description and sources is given in Table 1.

When integrating the above deterministic Eq (10) with respect to *t* we obtain trajectories of the mean behaviour which show damped oscillations tending to a fixed point see Fig 2. This is eventually the expected long-term behaviour for realistic parameter values for host-vector models. This further confirm the results of system stability analysis.

The stability of the steady state of this system is tractable, and can be obtained by deriving the deterministic limit (see subsection D of S1 Methods). It is easy to verify that these equations have a trivial fixed point, named the disease-free equilibrium *E*^0^:

$$\begin{array}{c} \phi_{1}^{0},\;\;\phi_{2}^{0},\;\;\psi^{0}; \end{array}$$

and a unique non-trivial fixed point named the endemic equilibrium E*:

$$\begin{array}{c} \phi_{1}^{*}=\frac{a+\mu_{2}R_{0}}{\left(a+\mu_{2}\right)R_{0}},\;\;\phi_{2}^{*}=\frac{\mu_{1}\mu_{2}\left(1-q_{1}\right)\left(R_{0}-1\right)}{b(a+\mu_{2})},\;\;\psi^{*}=\frac{\mu_{1}\mu_{2}g\left(1-q_{1}\right)\left(R_{0}-1\right)}{a(b\mu_{2}+\mu_{1}g\left(1-q_{1}\right))}, \end{array}$$

where *a* = *β*_21_*α*′ *m*_0_, *b* = *β*_12_*α*′, *g* = *ϵ*_2_ + *μ*_2_ and $R_{0}=\frac{1}{1-q_{1}}\frac{\beta_{21}\alpha^{\prime }m_{0}}{\mu_{1}}\frac{\beta_{12}\alpha^{\prime }}{\epsilon_{2}+\mu_{2}}$ is the basic reproductive number. From the stability’s analysis in Section D of S1 Methods, we know that when *R*_0_ < 1, the disease-free equilibrium point *E*^0^ is stable while when *R*_0_ > 1, the endemic equilibrium point E* exists and is stable.

### Periodicity of the stochastic host-vector model

A fundamental question is whether the existence of a stable fixed point in the deterministic system generates oscillations and multi-year periodicity in the corresponding stochastic system [34]. In order to investigate this and describe the stochastic fluctuations of the system by an analytical method, we introduce step operators which allow us to express the master Eq (4) in a more compact form which further facilitate the expansion of the system. Details are given in Section C.2 of S1 Methods, where it is shown that the resulting master equation can be written in a power series of $N_{1}^{-1/2}$ and $N_{2}^{-1/2}$ and the step operators in terms of the fluctuation variables *x*_1_, *x*_2_ and *x*_3_. Then, at next-to-leading order of the newly formed master equation (??) we obtain a linear Fokker–Planck equation for the fluctuation variables *x*_*l*_(*l* = 1, 2, 3),

$$\begin{array}{c} \frac{\partial \Pi }{\partial t}=-\sum_{k,l=1}^{3}A_{kl}\frac{\partial (x_{l}\Pi )}{\partial x_{k}}+\frac{1}{2}\sum_{k,l=1}^{3}B_{kl}\frac{\partial^{2}\Pi }{\partial x_{k}\partial x_{l}}. \end{array}$$ (11)

This is equivalent to a set of Langevin equations [42] for the stochastic corrections to the deterministic Eq (10) having the form

$$\begin{array}{c} \frac{dx_{k}}{dt}=\sum_{l=1}^{3}A_{kl}x_{l}+\xi_{k}\left(t\right),\;\;\left(k,l=1,2,3\right), \end{array}$$ (12)

where *ξ*_*k*_(*t*)(*k* = 1, 2, 3) are Gaussian white noises with zero mean and a cross-correlation function given by $\langle \xi_{k}\left(t\right)\xi_{l}\left(t^{\prime }\right)\rangle =B_{kl}\delta \left(t-t^{\prime }\right)$. Note that system Eq (12) combines both the deterministic and stochastic contributions. Given that we are interested in evaluating fluctuations of the system trajectories around the non-trivial fixed point of the deterministic system, we evaluate the entries of the Jacobian matrix *A*_*kl*_ and *B*_*kl*_ of the noise covariance matrix at this stable fixed point. Explicit expressions for these two matrices are given in subsection C.2 of S1 Methods.

The Langevin Eq (12) describe temporal evolution of the normalized fluctuations of variables around the equilibrium state. By Fourier transformation of these equations, we are able to analytically calculate the power spectral densities (PSD) that correspond to the normalized fluctuations, independent of community sizes *N*_1_ and *N*_2_. By taking the Fourier transform of Eq (12), we transform them into a linear system of algebraic equations, which can be solved. After taking averages, in the three expected power spectra of the fluctuations of susceptible livestock, infected livestock and infected *Aedes* mosquitoes around the deterministic stationary values we obtain:

$$\begin{array}{ccc} P_{S_{2}}\left(\omega \right) & = & \left\langle {|{\tilde{x}}_{1}\left(\omega \right)|}^{2}\right\rangle =\frac{B_{11}\omega^{4}+\Gamma_{S_{2}}\omega^{2}+\chi_{S_{2}}}{{\left|D(\omega )\right|}^{2}},\\ P_{I_{2}}\left(\omega \right) & = & \left\langle {|{\tilde{x}}_{2}\left(\omega \right)|}^{2}\right\rangle =\frac{B_{22}\omega^{4}+\Gamma_{I_{2}}\omega^{2}+\chi_{I_{2}}}{{\left|D(\omega )\right|}^{2}},\\ P_{I_{1}}\left(\omega \right) & = & \left\langle {|{\tilde{x}}_{3}\left(\omega \right)|}^{2}\right\rangle =\frac{B_{33}\omega^{4}+\Gamma_{I_{1}}\omega^{2}+\chi_{I_{1}}}{{\left|D(\omega )\right|}^{2}}, \end{array}$$ (13)

The complete derivation of these *PSDs* and detailed descriptions about the way the functions *χ*_*i*_, *B*_*kl*_, Γ_*k*_ and D(ω) depend on model parameters are discussed in subsection C.3 of S1 Methods.

## Results

### Probability of a major outbreak in the absence of vertical transmission

In the absence of vertical transmission, that is, *R*_11_ = 0 the solutions of the equations *G*_1_(*s*_1_, *s*_2_) = *s*_1_ and *G*_2_(*s*_1_, *s*_2_) = *s*_2_ are provided in [35] and for the case of introduction of a single infectious vector, it is reproduced here as follows:

To obtain the extinction probability requires determining the smallest non-negative root of

$$\begin{array}{c} s_{1}=\frac{1}{1+R_{21}\left[1-\frac{1}{1+R_{12}\left(1-s_{1}\right)}\right]}, \end{array}$$ (14)

which is obviously given by

$$\begin{array}{c} \frac{1+R_{12}}{R_{12}\left(R_{21}+1\right)}. \end{array}$$ (15)

**Fig 3:**
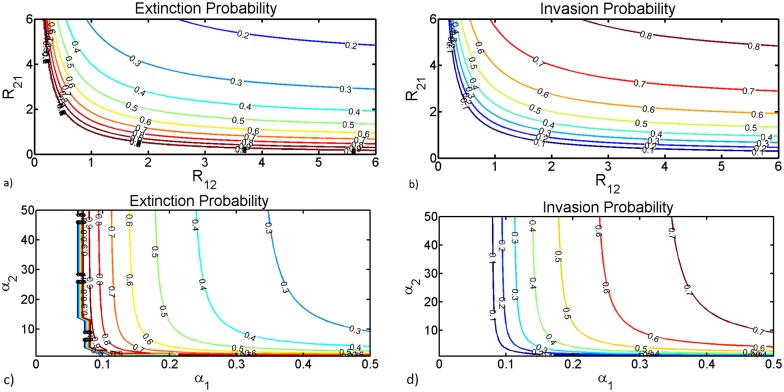
Solution of Eq (14) when the product *R*_12_ × *R*_21_ is greater than unity. The curves in (a) and (b) are contours in the plane (*R*_12_, *R*_21_), along which the probabilities of extinction and invasion respectively, after an introduction of a single vector is constant. In (c) and (d) we plot probabilities of extinction and invasion respectively, when varying parameters *α*_1_ and *α*_2_. The values of the remaining parameters in days are as follows: *μ*_1_ = 1/30, *μ*_2_ = 0.00046, *β*_12_ = 0.676, *β*_21_ = 0.28, *ϵ*_2_ = 0.25, *m*_0_ = 10.

Note that this is smaller than 1 if and only if the product *R*_12_*R*_21_ = *R*_0,*H*_ is greater than 1. Consequently, when *R*_0,*H*_ ≤ 1, the relevant solution is 1 and so a major outbreak can never happen [35, 63]. For *R*_0,*H*_ > 1, both the probability of extinction and of a major outbreak, are found by swapping the roles of *R*_12_ and *R*_21_ in the preceding elaboration. An interesting observation in host-vector systems is that *R*_0,*H*_ can be greater that one even if either *R*_12_ or *R*_21_ is less than unity. This leads to an asymmetry relationships between either with the probability of extinction or invasion and the reproductive numbers which may stem from the disparity between the sizes of the host and vector populations [35]. To further investigate this phenomenon we compute the probability of extinction and invasion while varying the biting ability of the vector when host ability to avoid a mosquito bite is taken into account. This is accomplished by varying the parameters *α*_1_ (number of bites that a mosquito would like to bite a host) and *α*_2_ (number of bites a host would sustain) when plotting the extinction and invasion probabilities. This is possible since in our approach we generalized the mosquito biting rates so that they can be applied to wider ranges of population sizes. Instead of letting the total number of mosquito bites on livestock depend on the number of mosquitoes as in [35], we set the total number of bites to vary with both the livestock and mosquito population sizes. Results from Fig 3(c) and 3(d) further rephrase the roots of the observed asymmetry highlighting that although the high ratio of mosquitoes to livestock is a major factor, any form of intervention to reduce livestock availability to mosquitoes can lead to such disparity. And disease extinction is only possible if the ratio mosquitoes to livestock is kept at a very low level resulting in values of *α*_1_ less than 0.1 see Fig 3(c). This explains why when environmental conditions are satisfied, that is, during rainy seasons disease outbreaks are expected as a result of the presence of massive numbers of potential vectors, implying large values of *α*_1_. From Fig 3(d) we see that for *α*_1_ around 0.5 invasion probabilities are close to 0.8. Hence, if mosquito biting activities are much more frequent disease invasion is expected but it is dependent on the availability of hosts. An interesting feature is that for *α*_1_ ≤ 1 invasion probability is zero regardless of the availability of hosts. This indicates that any intervention aimed at reducing the appetite of mosquitoes to bite might be a viable control strategy. Note also that the above observation may imply that infection does not die out merely because there are few susceptible hosts but because the number of infective vectors have reduced. Moreover, without virus reservoirs in either host or vector population or virus introduction from the outside even in the presence of optimal climatic conditions, disease activities are almost impossible. Therefore, in the following section we examine the relationships of disease persistence, extinction and spread when effects of vertical transmission efficiency are taken into consideration.

### Probability of a major outbreak in the presence of vertical transmission

In the presence of vertical transmission, determining the probability of extinction requires solving one of the equations in Eq (9) when *R*_11_ ≠ 0. In this regard, the extinction probability following the introduction of a single infectious mosquito is given by the smallest non-negative root [63] of

$$\begin{array}{c} s_{1}=\frac{1}{1+R_{11}\left(1-s_{1}\right)+R_{21}\left[1-\frac{1}{1+R_{12}\left(1-s_{1}\right)}\right]}. \end{array}$$ (16)

After rearranging the above equation we obtain

$$\begin{array}{c} R_{11}R_{12}{\left(1-s_{1}\right)}^{2}s_{1}+\left(R_{11}+R_{12}+R_{12}R_{21}\right)\left(1-s_{1}\right)s_{1}-R_{12}\left(1-s_{1}\right)+s_{1}-1=0, \end{array}$$ (17)

which is a cubic polynomial in *s*_1_. Note that for *R*_11_ = 0 this equation reduces to quadratic Eq (14). It is evident that *s*_1_ = 1 is a solution to Eq (17) and the remaining solutions are found by solving the quadratic equation

$$\begin{array}{c} R_{11}R_{12}s_{1}^{2}-\left(R_{11}+R_{12}+R_{11}R_{12}+R_{12}R_{21}\right)s_{1}+R_{12}+1=0. \end{array}$$ (18)

Denoting *A* = *R*_11_*R*_12_, *B* = *R*_11_ + *R*_12_ + *R*_11_*R*_12_ + *R*_12_*R*_21_ and *C* = *R*_12_ + 1, there exist a unique feasible solution to Eq (18) given by

$$\begin{array}{c} s_{1}=\frac{B-\sqrt{B^{2}-4AC}}{2A}, \end{array}$$

for more details see section B.3 of S1 Methods.

**Fig 4:**
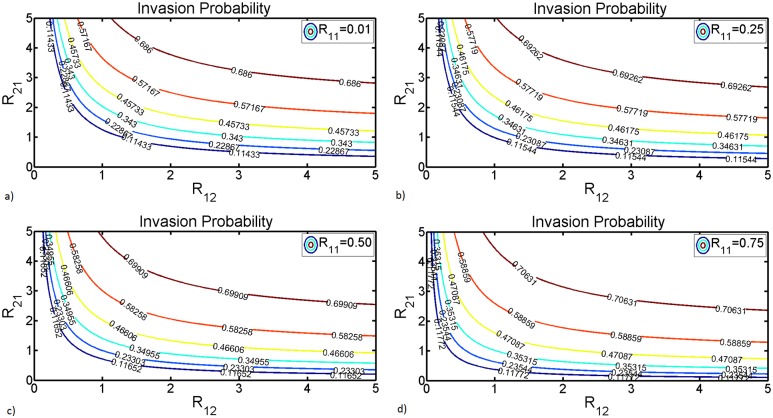
The curves represent contours in the plane (*R*_12_, *R*_21_), with varying vertical transmission efficiency, along which the probability of invasion after an introduction of a single vector is constant. These probabilities are obtained from the solutions of Eq (18).

**Fig 5:**
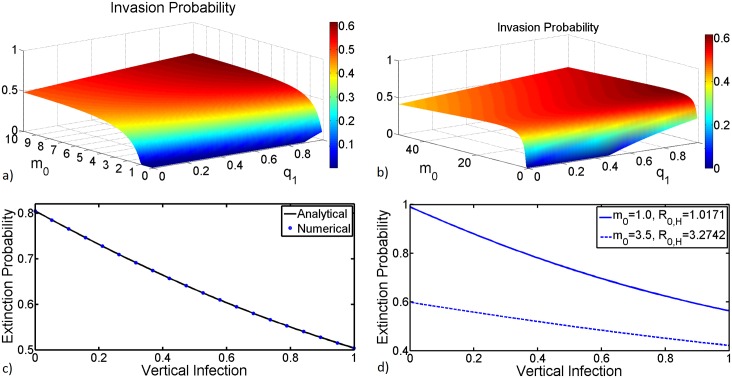
(a) and (b) A surface plot for the invasion probability when varying both vertical transmission, *q*_1_ and mosquitoes to hosts ratio, *m*_0_. (c) Numerical and analytical solution of the extinction probability Eq (18) when varying vertical transmission efficiency. (d) Analytical solutions of the extinction probability Eq (18) when varying vertical transmission efficiency for different values of the ratio female mosquitoes to hosts. The values of the remaining parameters in days are as follows: *μ*_1_ = 1/20, *μ*_2_ = 1/(8 ∗ 360), *β*_12_ = 0.55, *β*_21_ = 0.22, *ϵ*_2_ = 0.25 and *α*_1_ = 0.33, *α*_2_ = 19, *m*_0_ = 1.5 as baseline values.

Studies have shown that in the absence of vertical transmission in mosquitoes RVFV dies out when *R*_0_ < 1 and becomes endemic when *R*_0_ > 1. However, in the presence of vertical transmission the disease may persist even for *R*_0_ < 1 [24, 27, 28]. This situation stems from the fact that in host-vector systems, *R*_0_ results from a complete cycle of host-vector-host or vector-host-vector transmission and does not reflect the average number of secondary infections of a specific population type [69]. For instance, *R*_0_ = 0.75 may result from a product of host reproductive number *R*_12_ = 5 and vector reproductive number *R*_21_ = 0.15. Nevertheless, in each generation, the number of host infections is proportional to the number of infected mosquitoes, and decreases proportionally to the vertical infection efficiency. However, if the host reproductive number is high it is likely to boost up new vector infections in future generations. Fig 4 shows the dependency of probability of disease invasion on *R*_12_, *R*_21_ and vertical transmission efficiency *R*_11_. The invasion probability increases linearly with increments on vertical transmission efficiency with significant impact when vertical infection efficiency exceeds 20%. Other studies have found that it is only from such levels of vertical transmission efficiency that time of viral persistence is observed [69, 70]. Another interesting relationship is that as the invasion probability increases with vertical infection efficiency the horizontal transmission *R*_0,*H*_ = *R*_12_ × *R*_21_ tends to decrease highlighting an asymmetric relationship with *R*_12_ and *R*_21_ as highlighted in the previous section. Since one of the main confounding factors to such asymmetric relationship is the ratio female mosquitoes to hosts, we further investigate this phenomena by examining how both vertical transmission efficiency and ratio mosquitoes to hosts impact both the invasion and extinction probabilities. This is depicted in Fig 5 where we also provide a plot for both numerical and analytical solution of the extinction probability Eq (18) when varying vertical transmission efficiency. The results show that the invasion probability increases exponentially with respect to the ratio mosquitoes to hosts but increases linearly with respect to vertical transmission efficiency, Fig 5(a). However, it saturates when the ratio mosquitoes to hosts is close to *α*_2_, the number of bites a host would sustain, see Fig 5(b). This indicates that any adequate intervention aimed at preventing ruminants from being bitten is a viable control strategy regardless of the ratio mosquitoes to hosts. Since, Eq (18) is a polynomial of degree two its numerical and analytical solutions overlap and the extinction probability decreases quasi-linearly with respect to vertical infection, with the invasion lying above 0.5 Fig 5(c). This stems from the fact that the horizontal basic reproductive number, *R*_0,*H*_ is greater than unity, meaning that there are sustained host-to-vector and vice versa transmission cycles regardless of the efficiency of vertical transmission. A clear effect of the ratio mosquitoes to hosts is observed in Fig 5(b) where for very low vertical transmission efficiency and *m*_0_ = 1.0 the extinction probability is almost certain. This suggests that in the absence of vertical transmission, if every mosquito is for only one ruminant then there is a high probability that the disease will die out. This result from the fact that in such settings the chance of a ruminant being bitten twice in quick succession (once to catch the infection and once to pass it before recovery) is very small [59]. This is also depicted in (a) where for *m*_0_ ≤ 1 the invasion probability is almost null regardless of the efficiency of vertical transmission, but for $q_{1}⋙0.8$ invasion would be possible. More interestingly is the fact that for high ratios of female mosquitoes to hosts the level of vertical infection necessary for invasion decreases substantially Fig 5(b).

### Temporal patterns of Rift Valley fever in Sub-Saharan Africa

**Fig 6:**
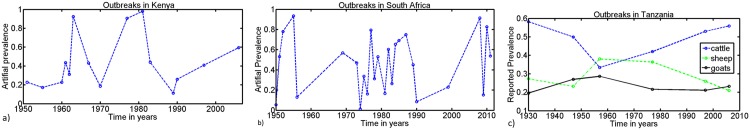
Temporal history of RVF outbreaks in some countries of Sub-Saharan Africa. In (a) and (b) the circles represent years of outbreaks occurrence in Kenya and South Africa [3, 9] and the prevalence indicated in the figure is not real, it is just for representation only since data on prevalence is not available. In (c) the circles represent the prevalence of disease outbreaks in Tanzania [7].

RVF is known to be endemic in Sub-Saharan Africa [14] with some differences in temporal patterns. In general it is emphasized that outbreaks occur at irregular intervals of up to 15 years in eastern and southern regions of the continent [7]. However, a closer look at temporal patterns of disease outbreaks in Tanzania and Kenya (East Africa) and South Africa (Southern Africa) shows existence of some possible differences in the temporal characteristic patterns of disease outbreaks. Fig 6 depicts temporal characteristic patterns of disease outbreaks from 1930 to 2007 in Tanzania [7], from 1951 to 2007 in Kenya [3] and from 1950 to 2011 in South Africa [9]. The prevalence shown for Kenya and South Africa is artificial, it is only for representation purposes since real information regarding prevalence of the disease at each year is not available. Although data regarding reported cases for each outbreak during the recent years may exist, it is not complete [2, 7]. For instance, in Tanzania, data for the years 1960, 1963 and 1968 is missing. The plots in Fig 6 are based on data reported in [3] for Kenya, in [9] for South Africa and in [7] for Tanzania. According to Pienaar and Thompson [9] during this period South Africa experienced only three major outbreaks (1950-1951, 1974-1976 and 2010-2011) and the remaining are considered smaller or isolated outbreaks. Interestingly the 1974 outbreak lasted for 3 consecutive years, a situation which can be compared to the 1960 outbreak that occurred in Kenya which continued until 1964 [3]. From the time series Fig 6(b) we observe that after each major outbreak including the outbreak in 1985-1986 in South Africa there are subsequent outbreaks occurring nearly each year. According to findings by Murithi et al. [3] during the period 1950-2007 only 11 large scale outbreaks were recorded in Kenya with an average inter-epizootic period of 3.6 years (range 1-7 years). However, for Tanzania an average inter-epizootic period of 7.9 years (range 3-17 years) is reported [7]. These disease post-epidemic activities in ruminants are known to occur without clinical cases and can only be detected where active surveillance is carried out [47, 71]. Could it be that these differences in temporal patterns are results of a deficit of surveillance system to cover all remote regions that are vulnerable to the disease or are due to differences in the ecology of the vector? This question takes us to another question which is the driving force of this study. Could it be possible that smaller or sporadic RVF outbreaks occur every year after major outbreaks without noticeable outbreaks or clinical cases due lack of active surveillance? Could the prevalence of these outbreaks show multi-year periodicity? If disease prevalence data could be available we would apply techniques of wavelet analysis which performs a time-scale decomposition of a time signal to estimate spectral characteristics of the signal as a function of time [31, 72]. This would allow us to predict the dominant period of outbreak fluctuations when varying some model parameters in particular, vertical transmission which is known to be the driving force behind the continuous disease endemicity in these regions [7]. Since reliable information is not available, in the following section we theoretically estimate the power spectra of disease oscillations taking into account effects of demographic stochasticity and vertical transmission.

### Effects of stochasticity and vertical transmission on disease outbreaks

**Fig 7:**
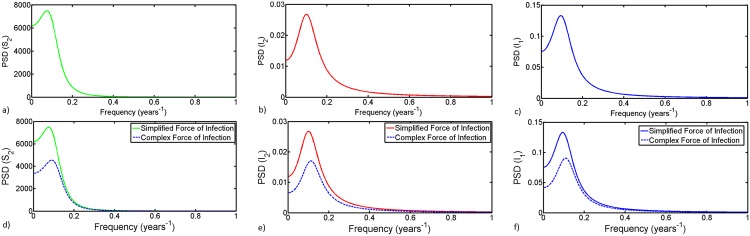
Theoretical prediction of the power spectrum density (PSD) (Eq (13)) for fluctutions of the total number of susceptible livestock, infected livestock and infected mosquitoes. (First Row) The theoretical prediction using the simplified force of infection. The values of the parameters used in years are as follows: *q*_1_ = 0.05, *μ*_1_ = (1/16) ∗ 360, *μ*_2_ = 1/8, *β*_12_ = 0.170, *β*_21_ = 0.116, *ϵ*_2_ = (1/4) ∗ 360, *α*′ = *α* = 256 and *m*_0_ = 1.5. This gives *R*_0_ = 1.0066. (Second Row) Comparison between theoretical predictions of PSD under the simplified and complex versions of the forces of infection. For the complex force of infection the new parameters are *α*_1_ = 0.33, *α*_2_ = 19, *m*_0_ = 9.45 and $\alpha^{\prime }=\frac{\alpha_{1}\alpha_{2}}{\alpha_{1}m_{0}+\alpha_{2}}$, and *R*_0_ = 1.0074. Note that description and sources of all model parameters are given in Table 1.

**Fig 8:**
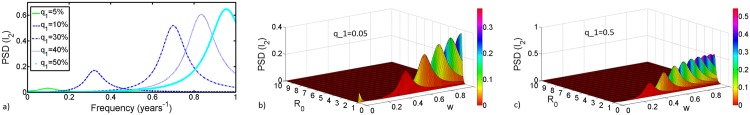
Power Spectra Density (PSD) for the variable *I*_2_ (Eq (13)). a) Effects of vertical transmission efficiency on the PSD. Three-dimensional representation of the PSD when varying *R*_0_ and the frequency for *q*_1_ = 0.05 and *q*_5_ = 0.5 in b) and c) respectively. Model parameter values used are as follows: *β*_12_ = 0.170, *β*_21_ = 0.116, *ϵ*_2_ = (1/4) ∗ 360, *α*′ = *α* = 256, *μ*_2_ = 1/8, *m*_0_ = 1.5, *μ*_1_ = (1/16) ∗ 360.

Fig 7 (first row) depicts the power spectrum density (PSD) for fluctuations of the total number of susceptible livestock, infected livestock and infected mosquitoes as derived in Eq (13), when using the standard or simplified version of the forces of infection. Our derivation of exact expressions for the power spectrum of the stochastic variables around the endemic equilibrium, see (Eq (13)) gives additional benefits. Using the expression for the power spectrum density (PSD) for variable *I*_2_ we examine how changes in female *Aedes* vertical transmission efficiency affects the periodicity of RVF outbreaks. In Fig 8(a) we observe that an increase in vertical transmission efficiency causes a significant increase in the frequency of disease outbreaks. To better illustrate this phenomenon, we show that for vertical transmission of *q*_1_ = 0.05 the dominant period of disease outbreaks is about 10 years while for *q*_1_ = 0.5 the dominant period is about 1 year. These results suggest that with low efficiency of vertical transmission there is a high probability of disease extinction after a major outbreak, followed by a long period without outbreaks. This stems from the fact that the mosquito life cycle is relatively short and vertically acquired infections are multiplicatively diluted with every generation such that the virus is rapidly lost unless there is regular amplification in the host population. This could be only possible if renewal of susceptible livestock would happen with high frequency. Since the PSD Formula (13) describes components of the deterministic model we can examine effects of the nature of the basic reproduction number *R*_0_ on outbreaks periodicity. If *R*_0_ is less than or equal to unity, with a high probability the disease outbreak is relatively small. This is the reason why most studies would rather concentrate on the complementary case. However, our analysis (see Fig 8(b) and 8(c)) shows that the most important and interesting case is where *R*_0_ is near unity. We see that as *R*_0_ moves away from unity the PSD surface becomes flatter, indicating that more frequencies are involved in the stochastic fluctuations. This simply means that when increasing *R*_0_, the dominant period decreases (the dominant frequency increases), however for larger values (*R*_0_ > 2) the PSD becomes totally flat. In this region ‘coherence resonance’, that is, a phenomenon in which random fluctuations sustain nearly periodic oscillations around the deterministic endemic equilibrium is lost and becomes white noise. Furthermore, we examine the PSD surface for nearly extreme values of vertical transmission efficiency *q*_1_ = 0.05 and *q*_1_ = 0.5. For larger values of vertical transmission the frequency of system fluctuation tends to increase, resulting in continuous endemicity of the disease as has been observed in some of the endemic regions [7]. While for small values of vertical infections the frequency of outbreaks is significantly reduced.

## Discussion

We have explored the use of analytical tools to measure and examine effects of demographic stochasticity in host-vector models with two routes of transmissions. Host-vector models are designed to explain the dynamics of diseases in which transmission of the pathogen is mediated by a vector. For our study case which is Rift Valley fever (RVF), the vector is a mosquito of genus *Aedes* with special ability of transmitting the virus to its offspring transovarially. In disease dynamics, this leads to two modes of transmission: horizontal and vertical. The later is of great epidemiological significance for it allows for investigating the contribution of this mode of transmission to disease spread and endemicity. The analytical tools applied are: branching process theory to examine the impact of stochastic effects on the invasion and persistence of RVF infection when vertical transmission is taken into account and the van Kampen method to investigate effects of mosquito vertical transmission on the characteristic temporal patterns of multi-year periodic disease outbreaks. Using branching process theory we have determined novel relationships among vertical infection, host-to-vector and vector-to-host reproductive numbers with both the invasion and extinction probabilities. These horizontal basic reproductive numbers are found to exhibit an asymmetric relationship with the probabilities of a major outbreak and extinction. Previous studies on host-vector models, using this technique highlighted that the existing asymmetry relationship between the disease transmission potentials from hosts to vectors and from vectors to hosts could stem from the fact that the disease invasion probability starting from a single infective host and the invasion probability starting from a single infective vector can differ significantly, even though the overall basic reproductive number of the infection is the same in both cases [35]. This asymmetry can lead to a situation where the overall basic reproduction number is greater than unity while either the vector or host reproductive number is less than unity, resulting in dramatic implications for disease control efforts. Unlike in previous models, we set the forces of infections to vary according to the sizes of both the host and vector populations. In this settings we further investigated the implications of this asymmetry relationships to disease control strategies by computing the invasion and extinction probabilities when varying the mosquito biting ability *α*_1_ and the host ability to avoid mosquito bites *α*_2_. Our model predictions suggest that although the ratio of mosquitoes to livestock is a major factor, any form of intervention to reduce livestock availability to mosquitoes can lead to such disparity.

Previous studies have shown that in the absence of vertical transmission in mosquitoes Rift Valley fever virus (RVFV) dies out when *R*_0_ < 1 and becomes endemic when *R*_0_ > 1. However, in the presence of vertical transmission the disease may persist even for *R*_0_ < 1 [24, 27, 28]. To further investigate the role played by this mode of transmission, for the first time using branching process theory we derive both the invasion and extinction probabilities on a host-vector model that includes vertical transmission. It has been shown for host-vector models without vertical transmission that in regard to invasion probability the two transmission potentials can show complex relationships, causing the invasion probability to remain almost constant as a given model parameter is varied. However, it is not the case of our model which has two routes of infection transmission. Our results suggested that invasion probability increases linearly with increments on vertical transmission efficiency with significant impact when vertical infection efficiency exceeded 20% as found in other studies of vector-borne diseases [69, 70]. Adams and Boots [69] found that vertical infection could only be important in dengue ecology, if the efficiency in nature is substantially greater than that found in empirical studies. On the contrary, vertically acquired infections are multiplicatively diluted at every mosquito life-cycle generation, such that, the virus is rapidly lost unless there is regular amplification in the host population. However, regular amplification of the virus in the host population is not certain for several factors. Recovered ruminants from RVF infection are immune for several days if not months [73], and vaccinated animals may produce a high level of neutralizing antibodies, making them protected against subsequent RVF viral infections [74]. However, how long do these neutralizing antibodies persist and other immune responses such as innate, humoral and cell mediated are not known with good degrees of certainty and require further investigation [2]. Another interesting factor is livestock renewal either through birth or migration, and the livestock viraemic phase whose intensity and duration may vary according to the inoculated dose, the virus strain and the degree of natural susceptibility of the infected ruminant [2]. Also, a factor that could serve as a constraint to regular amplification of the disease during the inter-epidemic period is the ratio mosquitoes to hosts (*m*_0_). For the first time we derived an explicit solution translating both the probability of major outbreak or extinction in a stochastic host-vector model with both horizontal and vertical transmissions. Our results showed that for *m*_0_ ≤ 1 the invasion probability is almost zero indicating that if mosquitoes are fewer compared to livestock, it is almost impossible for the infection to invade the community because sustained transmission may be impossible. An interesting pattern was observed when vertical transmission efficiency was in the range *q*_1_ ≫ 0.8, the disease could invade even for *m*_0_ ≤ 1. This finding suggest that the interplay between the two is also a determinant factor for disease spread and if not persistence. This interplay was more paramount for *m*_0_ > 1 where the levels of vertical transmission efficiency decreased substantially. This is another interesting finding in this paper, which highlights how interaction between the ratio mosquitoes to hosts and vertical infection efficiency influence both the invasion and extinction probabilities. In the case *m*_0_ > 1 there is a clear indication that during outbreak situation effects of vertical infection are easily diluted at every generation and this mode of transmission becomes more significant mostly at early stage of the epidemic. However, the invasion probability saturated for *m*_0_ close to *α*_2_ (host availability). Highlighting that if preventing measures targeting the host population are in place, the spread of infection will eventually saturate even for *m*_0_ ≫ 1 and higher level of vertical infection.

Results from experimental studies have indicated that depending on the host’s innate susceptibility or resistance the infection may be classified as: severe acute lethal infection, delayed onset of complications or mild to asymptomatic infection [75–77]. Low level asymptomatic circulation and host re-introduction from external reservoir populations are also likely to be important factors [24, 28, 69]. Chamchod et al. [27] concluded that re-introduction of susceptible animals from external sources (either through movement or buying) may lead to a certain probability of some subsequent outbreaks if the renewal takes place every year. Certainly in such a situation if vertical transmission is very low we are likely to observe long intervals with no outbreaks just like the situation in Tanzania (see Fig 6(a)); while for high values of vertical transmission we are likely to observe frequent waves of disease outbreaks as compared to the situation in South Africa Fig 6(b). Our results in Fig 4 further indicated that although invasion probability increases with vertical infection efficiency, the horizontal transmission reproductive number tends to decrease, highlighting an asymmetric relationship between the host and vector reproductive numbers. This further highlights the role of vertical transmission efficiency in inducing complex behaviours in the dynamics of RVF outbreaks. Such complex dynamics may partially be explained from the fact that effects of vertical infection are further compounded by effects of the diapause phenomena in *Aedes* mosquitoes [69], and the ratio female mosquitoes to livestock. In summary, our analysis reveals that higher values of vertical transmission or vertical infection efficiency increase the frequency of disease outbreaks and highlights the importance of the interplay between horizontal and vertical transmission [19, 24, 27, 28] in the spread and persistence of the disease.

Previous RVF modelling studies [24, 27, 28] have relied on the use of seasonal type functions in order to explain periodicity or subsequent waves of RVF outbreaks in endemic regions as well as characterizing the nature of the resulting oscillations when mosquito population varies according to seasons or climatic conditions [24, 27, 28]. This is the standard paradigm in the framework of deterministic models [31], where seasonal and/ or climatic extrinsic forcing and intrinsic host-pathogen dynamics are both used in order to understand the nature of different types of disease oscillations and system’s attractor structures [78]. However, more recently, it has become clear that the interaction between the deterministic dynamics and demographic stochasticity is fundamental to understand realistic patterns of disease outbreaks [30]. To the best of our knowledge this is the first time a non seasonal full stochastic host-vector model is used to explain the temporal characteristic patterns of disease multi-year periodicity depending on vertical transmission efficiency. This was accomplished by performing van Kampen [57] system size expansion, which allows us to derive an approximate analytical solution of the model. This method enables us to further view the population-level dynamics as being composed of a deterministic part and a stochastic part, where the spectrum of stochastic fluctuations is intimately related to the stability of the deterministic level dynamics [32]. Through power spectra analysis we were able to calculate the power spectrum of the stochastic fluctuations analytically and by comparison with simulations we can gain general insights into mechanisms underlying the peaks. Our analysis predicts complex fluctuations with a dominant period of 1 to 10 years for acceptable parameter values, which essentially depends on the efficiency of vertical transmission. Moreover, this dominant period was found to be significantly sensitive to the ratio mosquitoes to hosts and mosquitoes lifespan. These findings are in good agreement with observations, which indicate that in endemic areas RVFV is known to circulate continuously and outbreaks occur at irregular intervals of up to 15 years [3, 79], or 10-15 or even 3-7 years [3, 80]. Note however, that these periods of disease outbreaks are not known with exact details due to lack of appropriate infrastructure and active disease surveillance.

Although, we do not reproduce the exact known patterns of RVF outbreaks fluctuations in every country or region, we provide a plausible explanation, showing that the interplay between the stochastic component and vertical transmission is central to our understanding of the erratic patterns of disease outbreaks characterized by a dominant period of 1 to 10 years. Our results indicated that an increase in the vertical transmission efficiency increases the frequency of disease outbreaks, hence reducing the periodicity of outbreaks to nearly a dominant period of one year. This further confirms our findings through branching process theory as discussed above. When vertical infection efficiency is higher RVFV is likely to circulate every year with virus amplification at every rainfall season leading to yearly sporadic cases of disease outbreaks. This situation can be compared with the observation of disease outbreaks in South Africa as shown in Fig 6(b). According to a review by Pienaar and Thompson [9] since the first outbreak in 1950, South Africa has experienced only three major outbreaks (1950-1951, 1974-1976 and 2010-2011), with sporadic or isolated outbreaks in between. Two interesting temporal patterns can be discussed: (1) the post-epidemic disease activities or disease activities between two major outbreaks are of one year cycle; (2) the second major outbreak lasted for three consecutive years. Could it be that the efficiency of vertical transmission in South Africa is relatively higher, sustaining continuous endemicity patterns? Our analysis provides a simple but one of the most relevant explanations for this situation. An increase in vertical transmission efficiency leads to low frequency of disease outbreaks of nearly one year cycle which is in good agreement with findings from empirical studies [8, 9]. The epidemic continued through the winter, spilling over into the next rainfall season. It is believed that such spillover was possible due to warm temperatures and wet conditions during winter, which are conductive for reproduction of mosquitoes maintaining infection through winter. However, other dynamical factors such as susceptible livestock recruitment (or movement), mosquito seasonal abundance and livestock immune responses could play a role on fluctuations of RVF outbreaks [24, 27, 28]. Perhaps a combination of these factors was responsible for the 1974-1976 and 1960-1964 outbreaks in South Africa and Kenya respectively, which lasted for at least three consecutive years [3, 9]. Such ‘long-lasting’ consecutive outbreaks are not common and their underlying factors are not yet fully understood.

On the other hand, our model predicts that for low levels of vertical transmission the frequency of outbreaks becomes very low resulting in a dominant period of disease outbreaks of 10 years and above. These findings suggest that when efficiency of vertical transmission is very low the virus may require a long period of time to build up and eventually trigger an initial phase of the outbreak. This is a reasonable explanation for why there have been instances with no records of outbreaks following seasons of exceptionally above normal rainfall. This is likely to be the situation in East Africa, for example Tanzania (see Fig 6(a)). In this part of the continent outbreaks occur at irregular intervals followed by long periods (inter-epidemic period) without records of disease outbreaks, however, RVFV activities have been detected but with no clinical signs in the mammalian host [46, 47, 71]. During this inter-epidemic period (IEP) the virus exists but it fails to further amplify within the host during every wet season. Our explanation is that since the mosquito life cycle is very short, in the absence of regular amplification of the virus in the mammalian host population, vertically acquired infections can be rapidly lost. Low virus activities result in lower immunity in the host population and create conditions for large outbreaks whenever the virus may have sufficiently built up. In summary, for low vertical infection efficiency we expect long intervals without outbreaks. This is another contribution of this paper highlighting how our understanding of RVF ecology and epidemiology has been advanced by the work undertaken.

For a long time entomological studies have highlighted the relationship between abnormal rainfall and RVF outbreaks [3, 4, 10, 81]. However, optimum climatic conditions and the presence of mosquitoes have not completely explained the epidemiology of RVF outbreaks [82]. For instance, abundant rainfall, which normally correlates with increased number of mosquitoes in East Africa, was not often associated with RVF outbreaks in West Africa [2], and even in East Africa there have been instances where no outbreaks were recorded following seasons of exceptionally above normal rainfall [7]. These observations suggest that while rainfall might be the major determinant factor for the onset and switch-off of an outbreak [7], it is likely to not be the only factor responsible for the characteristic pattern of disease outbreaks. Other factors such as causal association between local environmental factors, livestock density and movement, encroachment of mosquitoes into new areas and livestock immune responses could be responsible for the observed characteristic pattern of disease outbreaks [7]. However, in this study we maintain the focus on the role of vertical transmission, ratio female mosquitoes to livestock and chance event on the oscillation of disease outbreaks and endemicity as we expect our results to be valid even when the above factors have been taken into account. Nevertheless, effects of livestock immune responses and livestock re-introduction or movement deserve their own further investigation.

## Supporting Information

S1 Methods Supporting methods. (PDF)
