# Supplementary material for: Predicting Rift Valley Fever Inter-epidemic Activities and Outbreak Patterns: Insights from a Stochastic Host-Vector Model
Source: PLoS Negl Trop Dis. 2016 Dec 21;10(12):e0005167. doi: 10.1371/journal.pntd.0005167 (PMC5176166; doi:10.1371/journal.pntd.0005167)
Supplement: S1 Methods — (PDF) [file pntd.0005167.s001.pdf]

Predicting Rift Valley fever Inter-Epidemic Activities and Outbreak Patterns: Insights from a Stochastic Host-Vector Model

Sansao A. Pedro[1,2,3,*], Shirley Abelman[1], Henri E.Z. Tonnang[4],

1 School of Computer Science and Applied Mathematics, University of the Witwatersrand, Johannesburg, Private Bag 3, Wits 2050, South Africa,
2 Modelling Unit, International Center of Insect Physiology and Ecology, 30772-00100, Nairobi, Kenya,
3 Departmento de Matemática e Informática, Universidade Eduardo Mondlane, 257, Maputo, Mozambique,
4 International Maize and Wheat Improvement Center (CIMMYT) ICRAF House, United Nation, Avenue, Gigiri, P.O. Box 1041 Village Market, 00621, Nairobi, Kenya.

* spedro@icipe.org/sansaopedro@gmail.com

Methods S1: Supporting Information

A Forces of Infection Approximation

Following the approach in Chitnis et al [1, 2] we derive disease forces of infection to be used in our model. Let α_1 be the rate at which a mosquito would want to bite a ruminant or the number of times one mosquito would want to bite a ruminant per unit time, defined as a function of its gonotrophic cycle. Let also α_2 be the maximum number o bites a particular ruminant can sustain per unit time. Thus, $\alpha_1 N_1$ gives the total number of bites that a mosquito would achieve per unit time, if ruminants were free available and $\alpha_2 N_2$ gives the availability of livestock. Assuming that the total number of mosquito-livestock contacts is defined as half the harmonic mean of $\alpha_1 N_1$ and $\alpha_2 N_2$,

$$a = a(N_1, N_2) = \frac{\alpha_1 N_1 \alpha_2 N_2}{\alpha_1 N_1 + \alpha_2 N_2}. \tag{A.1}$$

Hence, the number of bites per livestock per unit time and the number of bites per mosquito per unit time can be defined as

$$a_2 = a_2(N_1, N_2) = a(N_1, N_2)/N_2 = \frac{\alpha_1 \alpha_2 N_1}{\alpha_1 N_1 + \alpha_2 N_2}, \tag{A.2}$$

$$a_1 = a_1(N_1, N_2) = a(N_1, N_2)/N_1 = \frac{\alpha_1 \alpha_2 N_2}{\alpha_1 N_1 + \alpha_2 N_2}, \tag{A.3}$$

respectively. Therefore, the force of infection from mosquitoes to livestock, λ_{21}, can be defined as the product of the number of mosquito bites that a ruminant can sustain per unit time, a_2, the probability of successful infection transmission from an infected mosquito to a susceptible ruminant, β_{21}, and the prevalence of infectious mosquitoes, I_1/N_1. Then, λ_{21} is given as follows,

$$\lambda_{21} = \beta_{21} \frac{\alpha_1 \alpha_2 N_1}{\alpha_1 N_1 + \alpha_2 N_2} \frac{I_1}{N_1}. \tag{A.4}$$

The force of infection from livestock to mosquitoes, λ_{12}, can be defined as a product of the number of livestock bites one mosquito has per unit time, a_1, the probability of successful infection transmission from n infected ruminant to a susceptible mosquito, β_{12}, and the prevalence of the disease in livestock, I_2/N_2. Then, λ_{12} is given as follows,

$$\lambda_{12} = \beta_{12} \frac{\alpha_1 \alpha_2 N_2}{\alpha_1 N_1 + \alpha_2 N_2} \frac{I_2}{N_2}. \tag{A.5}$$

These forces of infections assume in principle that the total number of mosquito-livestock bites depend on the size of both population species. This formalism implies that both species contributes to the structure of contact rates mainly driven by the ratio mosquitoes to livestock and livestock availability to mosquitoes. However, if we are interested in the situation where mosquitoes bite hosts at a constant rate distributed uniformly among all hosts within an area, we obtain a frequency-dependent transmission mechanism with respect to the host population [3]. Thus, the above forces of infections λ_{21} and λ_{12} can be collapsed into simplified versions, by changing the contact structure,

$$a = a(N_1, N_2) = \frac{\alpha_1 N_1 \alpha_2 N_2}{\alpha_1 N_1 + \alpha_2 N_2} = \frac{\alpha_1 \alpha_2 N_1}{\alpha_1 (N_1/N_2) + \alpha_2}. \tag{A.6}$$

Since, we are interested on forces of infections in which contact rates or biting rates are expected to be constant irrespective of the number of available hosts, then it is reasonable to find the approximation as N_2 tends to infinity. That is,

$$a' = \lim_{N_2 \to \infty} a = \lim_{N_2 \to \infty} \frac{\alpha_1 \alpha_2 N_1}{\alpha_1 (N_1/N_2) + \alpha_2} = \alpha_1 N_1. \tag{A.7}$$

Hence, the corresponding number of bites per livestock per unit time is then given by $a'_2 = a'/N_2 = \alpha_1 N_1/N_2 = \alpha_1 m_0$, where m_0 is the ratio female mosquitoes to livestock. Similarly, the mosquito biting rate, that is, the number of bites per unit time is given by $a'_1 = a'/N_1 = \alpha_1$. Denoting, $\alpha_1 = \alpha$, yield the following standard forces of infections for mosquito transmitted diseases, $\lambda'_{21} = \beta_{21} \alpha m_0 \frac{I_1}{N_1}$ and $\lambda'_{12} = \beta_{12} \alpha \frac{I_2}{N_2}$.

B Stochastic Processes

B.1 Branching process approximation

Branching processes play a fundamental role in epidemic theory, underpinning our understanding of the threshold behaviour of epidemics and the calculation of both probability of disease extinction and invasion, while providing a simple way for modelling the spread of an infection at early stages of the epidemic [4, 5]. In multi-type branching process, individuals in the population are categorised into a finite number of types and each individual behaves independently. An individual of given type can produce offspring of possibly all types and individuals of the same type have the same offspring distribution [6, 7]. Given that infectious hosts and infectious vectors are the only sources of infection, the branching process is applied only to these infectious groups keeping the susceptibles at disease-free steady state [8]. Infectious vectors produce infected host when they bite a susceptible animal as well as infected vector through vertical transmission. Infectious hosts produce infected vectors when bitten by susceptible vector. Therefore, the number of infectives in the host-vector system during the early stages of the epidemic process is approximated by a two type branching process. Here infectious vectors are of type 1 and infectious hosts are of type 2. Note that first we consider the host-vector dynamics then later the vector-vector transmission.

B.2 Disease threshold conditions

Using the theory of multitype branching processes we have approximated the nonlinear stochastic model near the disease-free equilibrium (DFE). With this approximation we can now derive an estimate for the probability of disease extinction or a major outbreak after introduction of a single infective individual. Recall that we have two types of infective individuals: type 1 infective vectors and type 2 infective animals. Let $\{X_{ij}, i, j = 1, 2\}$ be the number of infectives of type j produced by an infective of type i and $m_{ij} = E[X_{ij}]$. We now derive the offspring distributions and expected numbers for the approximating branching process.

An infectious *Aedes* mosquito produces at most one single infectious host, but no other offspring, hence $X_{11} \equiv 0$. While on the ground an infectious *Aedes* either dies at rate μ_1 before surviving the intrinsic incubation period that is exponentially distributed with intensity γ_1, thus $P(X_{12} = 0) = \frac{\mu_1}{\gamma_1 + \mu_1}$. Or the infected *Aedes* mosquito survives the intrinsic incubation period with probability $\frac{\gamma_1}{\gamma_1 + \mu_1}$ and infects

a susceptible host according to a Poisson process with intensity $\frac{\alpha_1\alpha_2\beta_{21}}{\alpha_1 N_1+\alpha_2 N_2}$ within a period of time $T_1 = \frac{1}{\mu_1}$, since a mosquito once infected remains infected throughout its lifespan. For mathematical tractability this intermediate stage (incubation period) is not accounted for in this study.
Here we make the simplifying assumption that the number of infectious *Aedes* mosquitoes is very small, that is $I_1 = 1$.
Conditioning on T_1, the *Aedes* mosquito lifespan, the expected number of susceptible hosts that are infected before this period ends is

$$\begin{aligned} E[X_{12}] &= E(E[X_{12}|T_1]) = E\left(\frac{\alpha_1\alpha_2\beta_{21}}{\alpha_1 N_1+\alpha_2 N_2} S_2^0 T_1\right) = \frac{\alpha_1\alpha_2\beta_{21}}{\alpha_1 N_1+\alpha_2 N_2} S_2^0 E[T_1] \\ &= \frac{\alpha_1\alpha_2\beta_{21}}{\alpha_1 N_1+\alpha_2 N_2} S_2^0 \frac{1}{\mu_1} \end{aligned} \tag{B.1}$$

Next, an infectious host produces one infected *Aedes* mosquito if bitten by susceptible *Aedes* mosquito, hence $X_{22} \equiv 0$. A host is infectious for a time period that is exponentially distributed with intensity $\epsilon_2 + \mu_2$ (either it dies naturally at rate μ_2 or it recovers at the rate ϵ_2). During this period it infects susceptible *Aedes* according to a Poisson process with intensity $\frac{\alpha_1\alpha_2\beta_{12}}{\alpha_1 N_1+\alpha_2 N_2}$. Here we make the simplifying assumption that the number of infectious host is very small, that is $I_2 = 1$.
Thus, conditioning on $T_2 = \epsilon_2 + \mu_2$, the length of infectious period of a host, the expected number of susceptible *Aedes* that are infected before this period ends is

$$\begin{aligned} E[X_{21}] &= E(E[X_{21}|T_2]) = E\left(\frac{\alpha_1\alpha_2\beta_{12}}{\alpha_1 N_1+\alpha_2 N_2} S_1^0 T_2\right) = \frac{\alpha_1\alpha_2\beta_{12}}{\alpha_1 N_1+\alpha_2 N_2} S_1^0 E[T_2] \\ &= \frac{1}{\epsilon_2+\mu_2} \frac{\alpha_1\alpha_2\beta_{12}}{\alpha_1 N_1+\alpha_2 N_2} S_1^0. \end{aligned} \tag{B.2}$$

Let $\{m_{ij}\}_{ij=1}^2$ be the expected matrix of the form

$$M = \begin{pmatrix} 0 & \frac{\alpha_1\alpha_2\beta_{21}}{\alpha_1 N_1+\alpha_2 N_2} S_2^0 \frac{1}{\mu_1} \\ \frac{1}{\epsilon_2+\mu_2} \frac{\alpha_1\alpha_2\beta_{12}}{\alpha_1 N_1+\alpha_2 N_2} S_1^0 & 0 \end{pmatrix} \tag{B.3}$$

If the largest real-valued eigenvalue of M is less than or equal to unity, the epidemic dies out fairly quickly, while if the largest real-valued eigenvalue of M is greater than unity, there is a positive probability that the epidemic will take off [7].
The eigenvalues of M are the roots of the characteristic polynomial of matrix M. Since M is a regular matrix, it has all positive entries, then M has a positive eigenvalue λ that is larger than any other eigenvalue, which is given by

$$\lambda = \sqrt{\frac{\alpha_1\alpha_2\beta_{21}}{\alpha_1 N_1+\alpha_2 N_2} S_2^0 \frac{1}{\mu_1} \frac{1}{\epsilon_2+\mu_2} \frac{\alpha_1\alpha_2\beta_{12}}{\alpha_1 N_1+\alpha_2 N_2} S_1^0} \tag{B.4}$$

$R_{0,H} = \sqrt{R_{21}R_{12}}$ is the horizontal basic reproduction number, $R_{12} = \frac{1}{\epsilon_2+\mu_2} \frac{\alpha_1\alpha_2\beta_{12}}{\alpha_1 N_1+\alpha_2 N_2} S_1^0$ is the number of new infections in *Aedes* mosquitoes generated by single infected livestock and $R_{21} = \frac{\alpha_1\alpha_2\beta_{21}}{\alpha_1 N_1+\alpha_2 N_2} S_2^0 \frac{1}{\mu_1}$ is the number of new infections in livestock generated by single infected *Aedes* mosquito.
Since we are interested in the case where the largest eigenvalue is greater than unity, then $\lambda > 1$ implies that $\lambda^2 > 1$. This yields the following: $R_{0,H} = R_{21}R_{12}$.
We now relax the above assumption about transovarial transmission in *Aedes* mosquito species. Infectious female *Aedes* may infect their offspring during their life time which is exponentially distributed with intensity d_1, with the proportion $q_1 b_1$, hence the $P(X_{11} > 1) = q_1 b_1$ and the expected number of infected *Aedes* mosquitoes produced by a parent is

$$m_{11} = E[X_{11}] = \frac{q_1 b_1}{\mu_1} = q_1 \tag{B.5}$$

since at early stage of the epidemic the system is at equilibrium. Now, the threshold Γ can be written as

$$\Gamma = \frac{q_1}{2} + \frac{1}{2}\sqrt{q_1^2 + R_{0,H}^2} \tag{B.6}$$

Γ is the threshold quantity when the system is in equilibrium at the time of disease introduction. From equation (B.6) we notice that Γ has a monotonic dependence on all model parameters. When $\Gamma \leq 1$, the epidemic dies out fairly quickly since the probability of extinction is one and when $\Gamma > 1$, the epidemic may take off in the system and has a chance of becoming endemic since there is a positive probability of infection survival.

B.3 Probability of a major outbreak and disease extinction

With branching process theory we know that the likelihood of invasion depends not only on the average number of secondary infections (i.e. R_0), but also on their distribution [5]. Let the probability generating function of the offspring distribution of infectives produced by an infective of type $i(i = 1, 2)$, be $G_i(s) = E[\prod_{j=1}^{2} s_j^{X_{ij}}]$, where X_{ij} is as defined in the previous section and $s = (s_1, s_2)$.
The probability that a minor outbreak of the disease occurs given that there are a_j infectives initially of each of the two types is $\pi = \pi_1^{a_1}\pi_2^{a_2}$. Since M is irreducible, we know that $\pi_1 = \pi_2$ if $\Gamma \leq 1$ or that $\varphi(\pi_1, \pi_2)$ is the unique root of $s = G(s)$ that satisfies $\pi_1 < 1$ and $\pi_2 < 1$ if $\Gamma > 1$.
Since $X_{11} \equiv 0$ in the horizontal transmission and X_{12} is Poisson distributed conditioned on the infectious period $T_1 = t$ (as explained in the previous section), the probability generating function of offspring produced by one infected *Aedes* mosquito is

$$\begin{aligned} G_1(s) &= E[s_1^{X_{11}} s_2^{X_{12}}] = \sum_x s_2^x P(X_{12} = x) = \sum_x s_2^x \int_0^\infty \mu_1 e^{-\mu_1 t} \frac{e^{-g_3 N_2 t}(g_3 N_2 t)^x}{x!} dt \\ &= \mu_1 \int_0^\infty e^{-(\mu_1 + g_3 N_2)t} \left\{ \sum_{x=0}^\infty \frac{(g_3 N_2 s_2 t)^x}{x!} \right\} dt = \mu_1 \int_0^\infty e^{-(\mu_1+g_3N_2)t} e^{g_3 N_2 s_2 t} dt \\ &= \frac{\mu_1}{\mu_1 + g_3 N_2 - g_3 N_2 s_2} = \frac{1}{1 + R_{21}(1 - s_2)} \end{aligned} \tag{B.7}$$

Now in the presence of vertical transmission, that is, $X_{22} \neq 0$ and applying formula (4.8) in [5] we obtain that

$$G_1(s) = E[s_1^{X_{11}} s_2^{X_{12}}] = \frac{1}{1 + R_{11}(1 - s_1) + R_{21}(1 - s_2)}. \tag{B.8}$$

As $X_{22} \equiv 0$ and X_2 is Poisson distributed conditioned on the infectious period $T_2 = t$, the probability generating function of *Aedes* offspring produced by one infectious host is

$$\begin{aligned} G_2^1(s) &= E[s_1^{X_{21}}] = \sum_x s_1^x P(X_{21} = x) \\ &= \sum_x s_1^x \int_0^\infty (\epsilon_2 + \mu_2) e^{-(\epsilon_2+\mu_2)t} \frac{e^{-g_2 N_1 t}[g_2 N_1 t]^x}{x!} dt \\ &= (\epsilon_2 + m_2 + \mu_2) \int_0^\infty e^{-[\epsilon_2+\mu_2+g_2N_1]t} \left\{ \sum_{x=0}^\infty \frac{[g_2 N_1 s_1 t]^x}{x!} \right\} dt \\ &= (\epsilon_2 + \mu_2) \int_0^\infty e^{-[\epsilon_2+\mu_2+g_2N_1]t} e^{g_2 N_1 s_1 t} dt \\ &= \frac{\epsilon_2 + \mu_2}{\epsilon_2 + \mu_2 + g_2 N_1 - g_2 N_1 s_1} = \frac{1}{1 + R_{12}(1 - s_1)} \end{aligned} \tag{B.9}$$

In order to solve for extinction probabilities and probabilities of a major outbreak, we need to find the solution of the following system of two equations

$$G_1(s_1, s_2) = s_1 \texttt{ and } G_2(s_1, s_2) = s_2. \tag{B.10}$$

Given that we have a two-step life cycle from one type to another and then back to the original [5], the generating functions can be written as a composition function of the two single step generating functions [9]. Therefore, instead of solving equations (B.10) we solve the following equations:

$$G_1(G_2(s_1)) = s_1 \texttt{ and } G_2(G_1(s_1, s_2)) = s_2. \tag{B.11}$$

The vector $(s_1, s_2) = (1, 1)$ is always a solution. If $\Gamma \leq 1$ it is the only solution, whereas if $\Gamma > 1$ there are another solutions with all the components of the vector less than 1 [10], thus, the extinction probabilities are given by $\pi_i = min\{1, s_i\}$ for $i = 1, 2$.
In the presence of vertical transmission, the extinction probability following an introduction of a single infected mosquito is given by the smallest non-negative root of

$$s_1 = \frac{1}{1 + R_{11}(1 - s_1) + R_{21}[1 - \frac{1}{1+R_{12}(1-s_1)}]},$$

which can be written as

$$R_{11}R_{12}(1-s_1)^2 s_1 + (R_{11}+R_{12}+R_{12}R_{21})(1-s_1)s_1 - R_{12}(1-s_1) + s_1 - 1 = 0. \tag{B.12}$$

The above cubic polynomial has three possible solutions $1, s_1^1$ and s_1^2. The solutions s_1^1, s_1^2 are found by solving the quadratic equation

$$R_{11}R_{12}s_1^2 - (R_{11}+R_{12}+R_{11}R_{12}+R_{12}R_{21})s_1 + R_{12} + 1 = 0.$$

Lemma 1. *For $R_{0,H} > 1$, equation (B.12) has a unique feasible solution given by $s_1^2 = min\left\{1, s_1^1, s_1^2\right\}$.*

Proof. Clearly, unity is a trivial solution to equation (B.12). Now, let $A = R_{11}R_{12}$, $B = R_{11} + R_{12} + R_{11}R_{12} + R_{12}R_{21}$ and $C = R_{12} + 1$, then

$$s_1^1 = \frac{B + \sqrt{B^2 - 4AC}}{2A}, \quad s_1^2 = \frac{B - \sqrt{B^2 - 4AC}}{2A},$$

since

$$\begin{aligned} B^2 - 4AC &= (R_{11}+R_{12}+R_{11}R_{12}+R_{12}R_{21})^2 - 4R_{11}R_{12}(1+R_{12}), \\ &= R_{11}^2(1+R_{12})^2 + 2R_{11}R_{12}(1+R_{12})(-1+R_{21}) + R_{12}^2(1+R_{21})2, \\ &\geqslant R_{11}^2(1+R_{12})^2 - 2R_{11}R_{12}(1+R_{12})(1+R_{21}) + R_{12}^2(1+R_{21})2, \\ &\geqslant [R_{11}(1+R_{12}) - R_{12}(1+R_{21})]^2 > 0. \end{aligned}$$

Given that the discriminant is non-negative, then it is easy to see that $B + \sqrt{B^2 - 4AC}$ is always positive. Hence, to prove that s_1^2 is the feasible solution it is sufficient to check that $B - \sqrt{B^2 - 4AC}$ and $\frac{B-\sqrt{B^2-4AC}}{2A} < 1$. Clearly,

$$B - \sqrt{B^2 - 4AC} > 0 \Leftrightarrow B^2 > B^2 - 4AC \Leftrightarrow 0 > -4AC,$$

is greater than zero. Now, suppose $\frac{B-\sqrt{B^2-4AC}}{2A} > 1$, that is,

$$\begin{aligned} B - \sqrt{B^2 - 4AC} > 2A \Leftrightarrow A(B-C) < A^2 \Leftrightarrow B - C < A \\ \Leftrightarrow R_{11} + R_{12} + R_{11}R_{12} + R_{12}R_{21} - 1 - R_{12} < R_{11}R_{12} \\ \Leftrightarrow R_{11} + R_{12}R_{21} < 1. \end{aligned}$$

It is evident that for $R_{12}R_{21} > 1$ this leads to contradiction. Hence, $s_1^2 < 1$ and it is the extinction probability to our system with vertical transmission. □

C Analytical Analysis of the Stochastic Model

In this appendix we present an elegant mathematical formulation of the stochastic dynamics due to van Kampen's [11] system-size expansion method. In the first part we present the details of the mean-field version of the stochastic model. In the second part, the details of the calculation of the power spectral density from a stochastic Fokker-Planck equation. Then, stability analysis of the fixed points of the derived deterministic model.

C.1 The deterministic limit

First we write the master equation (4) in its generalized form

$$\begin{aligned} &\tfrac{dP(s_2,i_2,i_1;t)}{dt} = \\ &T(s_2,i_2,i_1|s_2+1,i_2-1,i_1)P(s_2+1,i_2-1,i_1;t) + T(s_2,i_2,i_1|s_2,i_2,i_1-1)P(s_2,i_2,i_1-1;t) \\ &+T(s_2,i_2,i_1|s_2-1,i_2,i_1)P(s_2-1,i_2,i_1;t) + T(s_2,i_2,i_1|s_2+1,i_2,i_1)P(s_2+1,i_2,i_1;t) \\ &+T(s_2,i_2,i_1|s_2,i_2,i_1+1)P(s_2,i_2,i_1+1;t) + T(s_2,i_2,i_1|s_2,i_2+1,i_1)P(s_2,i_2+1,i_1;t) \\ &-[T(s_2-1,i_2+1,i_1|s_2,i_2,i_1) + T(s_2,i_2,i_1+1|s_2,i_2,i_1) + T(s_2+1,i_2,i_1|s_2,i_2,i_1) \\ &+T(s_2-1,i_2,i_1|s_2,i_2,i_1) + T(s_2,i_2,i_1-1|s_2,i_2,i_1) + T(s_2,i_2-1,i_1|s_2,i_2,i_1)]P(s_2,i_2,i_1;t). \end{aligned} \tag{C.1}$$

This gives a complete description of the time evolution of the temporal model, from which we can obtain the deterministic analogues [12]. Following the notation and elaboration in [13], a straight forward way is to multiply (C.1) by s_2, i_2 and i_1 in turn and subsequently to sum over all allowed values of s_2, i_2 and i_1, and to take all the boundary values zero [12]. This gives equations for the mean $S_2 = \langle s_2 \rangle$, $I_2 = \langle i_2 \rangle$ and $I_1 = \langle i_1 \rangle$. For $S_2 = \langle s_2 \rangle = \sum_{s_2,i_2=0}^{N_2} \sum_{i_1=0}^{N_1} s_2 P(s_2, i_2, i_1; t)$, the mean-field theory takes the form

$$\begin{aligned} &\tfrac{dS_2}{dt} = \tfrac{d\langle s_2 \rangle}{dt} = \\ &\sum_{s_2,i_2=0}^{N_2} \sum_{i_1=0}^{N_1} T(s_2+1, i_2, i_1 | s_2, i_2, i_1) P(s_2, i_2, i_1; t) \\ &- \sum_{s_2,i_2=0}^{N_2} \sum_{i_1=0}^{N_1} [T(s_2-1, i_2+1, i_1 | s_2, i_2, i_1) + T(s_2-1, i_2, i_1 | s_2, i_2, i_1)] P(s_2, i_2, i_1; t) \end{aligned} \tag{C.2}$$

A similar way gives the equations for $I_2 = \langle i_2 \rangle$ and $I_1 = \langle i_1 \rangle$:

$$\frac{dI_2}{dt} = \frac{d\langle i_2 \rangle}{dt} = \sum_{s_2,i_2=0}^{N_2} \sum_{i_1=0}^{N_1} [T(s_2-1, i_2+1, i_1 | s_2, i_2, i_1) - T(s_2, i_2-1, i_1 | s_2, i_2, i_1)] P(s_2, i_2, i_1; t) \tag{C.3}$$

and

$$\frac{dI_1}{dt} = \frac{d\langle i_1 \rangle}{dt} = \sum_{s_2,i_2=0}^{N_2} \sum_{i_1=0}^{N_1} [T(s_2, i_2, i_1+1 | s_2, i_2, i_1) - T(s_2, i_2, i_1-1 | s_2, i_2, i_1)] P(s_2, i_2, i_1; t) \tag{C.4}$$

Given the derived equations (C.2)-(C.4), we now take the mean-field limits, $N_1, N_2 \to \infty$, which allows us to take the replacement $\langle i_1 s_2 \rangle = \langle i_1 \rangle \langle s_2 \rangle$ and $\langle i_2 (N_1 - i_1) \rangle = \langle i_2 \rangle \langle N_1 - i_1 \rangle$ [12,13]. When applying the following fractional variables

$$\phi_1 = \lim_{N_2 \to \infty} \frac{S_2}{N_2}, \quad \phi_2 = \lim_{N_2 \to \infty} \frac{I_2}{N_2}, \quad \psi = \lim_{N_1 \to \infty} \frac{I_1}{N_1} \tag{C.5}$$

yield the following set of deterministic equations:

$$\begin{aligned} \tfrac{d\phi_1}{dt} &= -\beta_{21} \alpha' m_0 \psi \phi_1 + \mu_2 (1 - \phi_1), \\ \tfrac{d\phi_2}{dt} &= \beta_{21} \alpha' m_0 \psi \phi_1 - (\epsilon_2 + \mu_2) \phi_2, \\ \tfrac{d\psi}{dt} &= \beta_{12} \alpha' \phi_2 (1 - \psi) + \mu_1 q_1 \psi - \mu_1 \psi. \end{aligned} \tag{C.6}$$

C.2 The system-size expansion and analysis of the fluctuations

van Kampen's system expansion method [11], is an appropriate technique for characterizing disease fluctuations in order to investigate the effects of stochasticity in our model and for finding stochastic corrections to the resulting deterministic equations for large N_1 and N_2 [12]. To do so, we transform the stochastic discrete variables $\sigma = (s_2, i_2, i_1)$ to depend into new stochastic variables $\zeta = (x_1, x_2, x_3)$ as follows:

$$\begin{aligned} s_2 &= N_2 \phi_1 + \sqrt{N_2} x_1, \\ i_2 &= N_2 \phi_2 + \sqrt{N_2} x_2, \\ i_1 &= N_1 \psi + \sqrt{N_1} x_3. \end{aligned}$$

Then, the probability distribution $P(s_2, i_2, i_1; t)$ is now written as a function of the new variables x_1, x_2, x_3 as follows:

$$\frac{dP}{dt} = \frac{\partial \Pi}{\partial t} - \sqrt{N_2} \frac{d\phi_1}{dt} \frac{\partial \Pi}{\partial x_1} - \sqrt{N_2} \frac{d\phi_2}{dt} \frac{\partial \Pi}{\partial x_2} - \sqrt{N_1} \frac{d\psi}{dt} \frac{\partial \Pi}{\partial x_3}. \tag{C.7}$$

Before going further, we introduce an operator in the following form

$$\varepsilon_{\zeta}^{\pm 1} = 1 \pm \frac{1}{\sqrt{N_2}}\frac{\partial}{\partial \zeta} + \frac{1}{2N_2}\frac{\partial^2}{\partial \zeta^2},$$

which yield the following step operators [11,12],

$$\begin{aligned}
\varepsilon_{s_2}^{\pm 1} f(s_2, i_2, i_1) &= f(s_2 \pm 1, i_2, i_1),\\
\varepsilon_{i_2}^{\pm 1} f(s_2, i_2, i_1) &= f(s_2, i_2 \pm 1, i_1),\\
\varepsilon_{i_1}^{\pm 1} f(s_2, i_2, i_1) &= f(s_2, i_2, i_1 \pm 1),
\end{aligned}$$

which are then used to rewrite the master equation (C.1) with transition rates of equations $(1-3)$ as

$$\begin{aligned}
&\tfrac{dP(s_2,i_2,i_1;t)}{dt} = \\
&[(\varepsilon_{s_2}\varepsilon_{i_2}^{-1} - 1)T(s_2 - 1, i_2 + 1, i_1|s_2, i_2, i_1) + (\varepsilon_{i_1}^{-1} - 1)T(s_2, i_2, i_1 + 1|s_2, i_2, i_1)\\
&+(\varepsilon_{s_2}^{-1} - 1)T(s_2 + 1, i_2, i_1|s_2, i_2, i_1) + (\varepsilon_{s_2} - 1)T(s_2 - 1, i_2, i_1|s_2, i_2, i_1)\\
&+(\varepsilon_{i_1} - 1)T(s_2, i_2, i_1 - 1|s_2, i_2, i_1) + (\varepsilon_{i_2} - 1)T(s_2, i_2 - 1, i_1|s_2, i_2, i_1)]P(s_2, i_2, i_1; t)\\
&= \Big\{(\varepsilon_{s_2}\varepsilon_{i_2}^{-1} - 1)\beta_{21}\alpha' m_0 i_1 s_2 + (\varepsilon_{i_1}^{-1} - 1)[\beta_{12}\alpha' i_2(N_1 - i_1) + \mu_1 q_1 i_1]\\
&+(\varepsilon_{s_2}^{-1} - 1)\mu_2 N_2 + (\varepsilon_{s_2} - 1)\mu_2 s_2 + (\varepsilon_{i_2} - 1)(\epsilon_2 + \mu_2) i_2 + (\varepsilon_{i_1} - 1)\mu_1 i_1\Big\}P(s_2, i_2, i_1; t).
\end{aligned} \tag{C.8}$$

Expanding the step operators $\varepsilon_{s_2}^{\pm 1}$ and $\varepsilon_{i_2}^{\pm 1}$ in a power series in $N_2^{-1/2}$, $\varepsilon_{i_1}^{\pm 1}$ in $N_1^{-1/2}$, respectively,

$$\begin{aligned}
\varepsilon_{s_2}^{\pm 1} &= 1 \pm \frac{1}{\sqrt{N_2}}\frac{\partial}{\partial x_1} + \frac{1}{2N_2}\frac{\partial^2}{\partial x_1^2},\\
\varepsilon_{i_2}^{\pm 1} &= 1 \pm \frac{1}{\sqrt{N_2}}\frac{\partial}{\partial x_2} + \frac{1}{2N_2}\frac{\partial^2}{\partial x_2^2},\\
\varepsilon_{i_1}^{\pm 1} &= 1 \pm \frac{1}{\sqrt{N_1}}\frac{\partial}{\partial x_3} + \frac{1}{2N_1}\frac{\partial^2}{\partial x_3^2},
\end{aligned}$$

and then substituting these operators into equation (C.8), we get

$$\begin{aligned}
&\tfrac{dP}{dt} = \\
&\Big\{\Big[\Big(1 + \tfrac{1}{\sqrt{N_2}}\tfrac{\partial}{\partial x_1} + \tfrac{1}{2N_2}\tfrac{\partial^2}{\partial x_1^2}\Big)\Big(1 - \tfrac{1}{\sqrt{N_2}}\tfrac{\partial}{\partial x_2} + \tfrac{1}{2N_2}\tfrac{\partial^2}{\partial x_2^2}\Big) - 1\Big]\beta_{21}\alpha' m_0 i_1 s_2 + \Big(-\tfrac{1}{\sqrt{N_1}}\tfrac{\partial}{\partial x_3} + \tfrac{1}{2N_1}\tfrac{\partial^2}{\partial x_3^2}\Big)\\
&[\beta_{12}\alpha' i_2(N_1 - i_1) + \mu_1 q_1 i_1] + \Big(-\tfrac{1}{\sqrt{N_2}}\tfrac{\partial}{\partial x_1} + \tfrac{1}{2N_2}\tfrac{\partial^2}{\partial x_1^2}\Big)\mu_2 N_2 + \Big(\tfrac{1}{\sqrt{N_2}}\tfrac{\partial}{\partial x_1} + \tfrac{1}{2N_2}\tfrac{\partial^2}{\partial x_1^2}\Big)\mu_2 s_2\\
&+\Big(\tfrac{1}{\sqrt{N_2}}\tfrac{\partial}{\partial x_2} + \tfrac{1}{2N_2}\tfrac{\partial^2}{\partial x_2^2}\Big)(\epsilon_2 + \mu_2) i_2 + \Big(\tfrac{1}{\sqrt{N_1}}\tfrac{\partial}{\partial x_3} + \tfrac{1}{2N_1}\tfrac{\partial^2}{\partial x_3^2}\Big)\mu_1 i_1\Big\}\Pi(x_1, x_2, x_3; t).
\end{aligned} \tag{C.9}$$

Then, expanding Eq.C.9 and ignoring higher order terms and drawing a comparison of which with equation (C.7) order by order yields the so-called macroscopic equations

$$\frac{d\phi}{dt} = f_1(\phi_1, \phi_2, \psi), \qquad \frac{d\phi_2}{dt} = f_2(\phi_1, \phi_2, \psi), \qquad \frac{d\psi}{dt} = (\phi_1, \phi_2, \psi), \tag{C.10}$$

to leading order, where

$$\begin{aligned}
f_1(\phi_1, \phi_2, \psi) &= -\beta_{21}\alpha' m_0 \psi \phi_1 + \mu_2(1 - \phi_1),\\
f_2(\phi_1, \phi_2, \psi) &= \beta_{21}\alpha' m_0 \psi \phi_1 - (\epsilon_2 + \mu_2)\phi_2,\\
f_3(\phi_1, \phi_2, \psi) &= \beta_{12}\alpha' \phi_2(1 - \psi) + \mu_1 q_1 \psi - \mu_1 \psi,
\end{aligned} \tag{C.11}$$

which are indeed the equations (C.6). The next-to-leading order gives rise to a Fokker-Planck equation for the fluctuation variables x_1, x_2, x_3

$$\frac{\partial \Pi}{\partial t} = -\sum_{k,l=1}^{3} A_{kl}\frac{\partial(x_l \Pi)}{\partial x_k} + \frac{1}{2}\sum_{k,l=1}^{3} B_{kl}\frac{\partial^2 \Pi}{\partial x_k \partial x_l}. \tag{C.12}$$

To obtain the coefficients A_{kl} and B_{kl} we expand the equation (C.12) and draw comparison order by order with equation (C.9). Since we are interested in fluctuations about the endemic equilibrium point $E^* = (\phi_1^*, \phi_2^*, \psi^*)$ defined in equation (C.6) of the deterministic model, both matrix $\mathcal{A} = (A_{kl})_{3\times3}$ and $\mathcal{B} = (B_{kl})_{3\times3}$ are evaluated at this fixed point, whose explicit form are found to be

$$\mathcal{A} = \begin{pmatrix} \frac{\partial f_1}{\partial \phi_1} & 0 & \sqrt{1/m_0}\frac{\partial f_1}{\partial \psi} \\ \frac{\partial f_2}{\partial \phi_1} & \frac{\partial f_2}{\partial \phi_2} & \sqrt{1/m_0}\frac{\partial f_2}{\partial \psi} \\ 0 & \sqrt{m_0}\frac{\partial f_3}{\partial \phi_2} & \frac{\partial f_3}{\partial \psi} \end{pmatrix}_{\phi_1=\phi_1^*, \phi_2=\phi_2^*, \psi=\psi^*} \quad \text{and } \mathcal{B} = \begin{pmatrix} B_{11} & B_{12} & 0 \\ B_{21} & B_{22} & 0 \\ 0 & 0 & B_{33} \end{pmatrix}_{\phi_1=\phi_1^*, \phi_2=\phi_2^*, \psi=\psi^*} \tag{C.13}$$

with

$$\begin{aligned} B_{11} &= \beta_{21}\alpha' m_0 \psi\phi_1 + \mu_2(1+\phi_1), \\ B_{12} &= B_{21} = -2\beta_{21}\alpha' m_0\psi\phi_1, \\ B_{22} &= \beta_{21}\alpha' m_0\psi\phi_1 + (\epsilon_2+\mu_2)\phi_2, \\ B_{33} &= \beta_{12}\alpha'\phi_2(1-\psi) + \mu_1 q_1\psi + \mu_1\psi. \end{aligned}$$

C.3 Power spectral calculation and its peak

To calculate the power spectra of the fluctuations around the stationary state, we have to make a Fourier analysis, so it is first essential to formulate a set of Langevin equations of the stochastic variables $x_k(t), (k = 1, 2, 3)$. The Langevin equations corresponding to equation (C.12) are

$$\frac{dx_k}{dt} = \sum_{l=1}^{3} A_{kl}x_l + \xi_k(t), \quad (k, l = 1, 2, 3) \tag{C.14}$$

which are three differential equations describing the stochastic behaviour of the model at large but finite N. The variables $x_l (l = 1, 2, 3)$ are stochastic corrections to the deterministic variables s_2, i_2, i_1, and $\xi_k(t)(k = 1, 2, 3)$ are Gaussian white noises with zero mean and a correlation function given by

$$\langle \xi_k(t)\xi_l(t')\rangle = B_{kl}\delta(t-t').$$

Taking the temporal Fourier transform $\tilde{x}_k(\omega) = \int_{-\infty}^{\infty} e^{-i\omega t}x_k(t)dt$ of (C.14) gives

$$-i\omega\tilde{x}_k(\omega) = \sum_{l=1}^{3} A_{kl}\tilde{x}_l(\omega) + \tilde{\xi}_k(\omega), \quad (k, l = 1, 2, 3) \tag{C.15}$$

with

$$\langle \tilde{\xi}_k(\omega)\tilde{\xi}_l(\omega')\rangle = B_{kl}(2\pi)\delta(\omega+\omega').$$

Actually, this Fourier transform is a system with three coupled linear algebraic equations which can be used to obtain a closed form expression for the power spectra. Therefore, solving equation (C.15), we obtain

$$\begin{aligned} \tilde{x}_1(\omega) &= \frac{(A_{23}A_{32}-A_{22}A_{33})\tilde{\xi}_1 - A_{13}A_{32}\tilde{\xi}_2 + A_{13}A_{22}\tilde{\xi}_3 + \omega^2\tilde{\xi}_1 + i\omega[-(A_{22}+A_{33})\tilde{\xi}_1 + A_{13}\tilde{\xi}_3]}{\mathcal{D}(\omega)}, \\ \tilde{x}_2(\omega) &= \frac{A_{21}A_{33}\tilde{\xi}_1 - A_{11}A_{33}\tilde{\xi}_2 + (A_{11}A_{23}-A_{21}A_{13})\tilde{\xi}_3 + \omega^2\tilde{\xi}_2 + i\omega[A_{21}\tilde{\xi}_1 - (A_{11}+A_{33})\tilde{\xi}_2 + A_{23}\tilde{\xi}_3]}{\mathcal{D}(\omega)}, \\ \tilde{x}_3(\omega) &= \frac{A_{21}A_{32}\tilde{\xi}_1 + A_{11}A_{32}\tilde{\xi}_2 - A_{11}A_{22}\tilde{\xi}_3 + \omega^2\tilde{\xi}_3 + i\omega[A_{32}\tilde{\xi}_2 - (A_{11}+A_{22})\tilde{\xi}_3]}{\mathcal{D}(\omega)} \end{aligned} \tag{C.16}$$

where the denominator $\mathcal{D}$ is given by

$$\begin{aligned} \mathcal{D}(\omega) &= (i\omega)^3 + tr\mathcal{A}(i\omega)^2 + \Theta(i\omega) + det\mathcal{A}, \\ tr\mathcal{A} &= A_{11} + A_{22} + A_{33}, \\ \Theta &= A_{11}A_{22} + A_{11}A_{33} + A_{22}A_{33} - A_{23}A_{32}, \\ det\mathcal{A} &= A_{11}A_{22}A_{33} - A_{11}A_{23}A_{32} + A_{21}A_{13}A_{32}. \end{aligned}$$

Averaging the squared moduli of $\tilde{x}_k(k=1,2,3)$ gives the power-spectra of variables S_2, I_2 and I_1:

$$\begin{aligned} P_{S_2}(\omega) &= \langle|\tilde{x}_1(\omega)|^2\rangle = \frac{B_{11}\omega^4+\Gamma_{S_2}\omega^2+\chi_{S_2}}{|\mathcal{D}(\omega)|^2}, \\ P_{I_2}(\omega) &= \langle|\tilde{x}_2(\omega)|^2\rangle = \frac{B_{22}\omega^4+\Gamma_{I_2}\omega^2+\chi_{I_2}}{|\mathcal{D}(\omega)|^2}, \\ P_{I_1}(\omega) &= \langle|\tilde{x}_3(\omega)|^2\rangle = \frac{B_{33}\omega^4+\Gamma_{I_1}\omega^2+\chi_{I_1}}{|\mathcal{D}(\omega)|^2}, \end{aligned} \tag{C.17}$$

where

$$\begin{aligned} |\mathcal{D}(\omega)|^2 &= (\omega^3-\Theta\omega)^2 + (det\mathcal{A} - tr\mathcal{A}\omega^2)^2, \\ \chi_{S_2} &= (A_{23}A_{32}-A_{22}A_{33})^2B_{11} - 2A_{13}A_{32}(A_{23}A_{32}-A_{22}A_{33})B_{12} + (A_{13}A_{32})^2B_{22} \\ &\quad + (A_{13}A_{22})^2B_{33}, \\ \Gamma_{S_2} &= 2(A_{23}A_{32}-A_{22}A_{33})B_{11} - 2A_{13}A_{32}B_{12} + (A_{22}+A_{33})^2B_{11} + A_{13}^2B_{33}, \\ \chi_{I_2} &= (A_{21}A_{33})^2B_{11} + (A_{11}A_{33})^2B_{22} + (A_{11}A_{23}-A_{21}A_{13})^2B_{33} - 2A_{11}A_{21}A_{33}^2B_{12}, \\ \Gamma_{I_2} &= 2A_{21}A_{33}B_{12} - 2A_{11}A_{33}B_{22} + A_{21}^2B_{11} - 2A_{21}(A_{11}+A_{33})B_{12} + (A_{11}+A_{33})^2B_{22} \\ &\quad + A_{32}^2B_{33}, \\ \chi_{I_1} &= (A_{21}A_{32})^2B_{11} + (A_{11}A_{32})^2B_{22} + (A_{11}A_{22})^2B_{33} + 2A_{11}A_{21}A_{32}^2B_{12}, \\ \Gamma_{I_1} &= A_{32}^2B_{22} + (A_{11}+A_{22})^2B_{33} - 2A_{11}A_{22}B_{33}. \end{aligned}$$

By using these methods, we can analytically predict the epidemic outbreaks and fade-outs on a certain disease, as done for several childhood diseases [14].

D Stability analysis of fixed points E^0 and E^* of system (C.6)

First, we give the stability analysis of the disease-free equilibria $E^0=(1,0,0)$, the Jacobian matrix of which is given by

$$J^0 = \begin{bmatrix} -\mu_2 & 0 & -a \\ 0 & -g & a \\ 0 & b & -\mu_1(1-q_1) \end{bmatrix} \tag{D.1}$$

where $a=\beta_{21}\alpha' m_0$, $b=\beta_{12}\alpha'$, $g=\epsilon_2+\mu_2$.
From the first column of the Jacobian matrix (D.1) we observe that the matrix has the eigenvalue $\lambda_1=\mu_2$, and the remaining eigenvalues will be derived from the reduced 2×2 Jacobian matrix $J_1^0 = \left[\begin{smallmatrix} -g & a \\ b & -\mu_1(1-q_1)\end{smallmatrix}\right]$, from which we obtain the trace, $tr(J_1^0) = -g-\mu_1(1-q_1)$, which is negative and its determinant is $det(J_1^0) = g\mu_1(1-q_1) - ab$.
Since for local stability it is sufficient to have $tr(J_1^0)<0$ and $det(J_1^0)>0$, so for determinant to be positive, we need to show that $g\mu_1(1-q_1)-ab>0$, that is $1-\frac{ab}{g\mu_1(1-q_1)}>0$. Thus, $\frac{ab}{g\mu_1(1-q_1)}<1$, which is the condition for basic reproduction number, i.e., $R_0<1$, and $R_0 = \frac{1}{1-q_1}\frac{\beta_{21}\alpha' m_0}{\mu_1}\frac{\beta_{12}\alpha'}{\epsilon_2+\mu_2}$.
Second, the stability analysis of the endemic equilibria $E^*=(\phi_1^*,\phi_2^*,\psi^*)$, with $\phi_1^* = \frac{\mu_2 R_0 + a}{(a+\mu_2)R_0}$, $\phi_2^* = \frac{\mu_1\mu_2(1-q_1)(R_0-1)}{b(a+\mu_2)}$, $\psi^* = \frac{\mu_1\mu_2 g(1-q_1)(R_0-1)}{a(b\mu_2+\mu_1 g(1-q_1))}$.
The Jacobian matrix at E^* is

$$J^* = \begin{bmatrix} -\frac{a\mu_2(R_0-1)}{fh} - \mu_2 & 0 & -\frac{af}{bl} \\ \frac{a\mu_2(R_0-1)}{fh} & -g & \frac{af}{bl} \\ 0 & b\left(1-\frac{\mu_2(R_0-1)}{fh}\right) & -\frac{b\mu_2(R_0-1)}{lm} - \mu_1(1-q_1) \end{bmatrix}$$

with $f=b\mu_2+\mu_1 g(1-q_1)$, $m=\frac{1}{1-q_1}\frac{b}{\mu_1}$, $h=\frac{1}{1-q_1}\frac{a}{g}$ and $l=a+\mu_2$.
The characteristic polynomial of Jacobian matrix J^* is written as cubic polynomial about λ denoted as $P(\lambda)$:

$$P(\lambda) = \lambda^3 + A\lambda^2 + B\lambda + C, \tag{D.2}$$

all of the coefficients of which are

$$A = \frac{fhb\mu_2(R_0-1) + fhlm\mu_1(1-q_1) + lma\mu_2(R_0-1) + lmgfh + lmfh\mu_2}{lmfh} > 0 \quad \text{for} \quad R_0 > 1,$$

$$B = \frac{1}{lmfh}[amf\mu_2(R_0-1) + bgfh\mu_2(R_0-1) + ab\mu_2^2(R_0^2 - 2R_0) + bfh\mu_2^2(R_0-1) + ab\mu_2^2 + lmgfh(1-q_1) + alm\mu_1\mu_2(R_0-1)(1-q_1) + lmfh\mu_1\mu_2(1-q_1)lmag\mu_2(R_0-1) + glmfh\mu_2] > 0 \quad \text{for} \quad R_0 > 1,$$

$$C = \frac{\mu_2}{lmfh}\{gab\mu_2(R_0^2 - 2R_0) + bgfh\mu_2(R_0-1) + glma\mu_1(R_0-1)(1-q_1) + lmgfh\mu_1(1-q_1) + amf[\mu_2(R_0-1) - fh]\}.$$

$C > 0$ if and only if $R_0 > 1$, given that $\psi^* > 0$. Here $A > 0, B > 0$ and $C > 0$ for $R_0 > 1$. Thus equation (D.2) has no root which is positive or zero (Descartes' rule of sign). The equation (D.2) will only have negative roots or complex roots with negative real part if $AB - C > 0$ according to the (Routh-Hurwitz criteria). Thus the system is stable about the interior equilibrium point E^* whenever it exists and $AB - C > 0$.

References

[1] N. Chitnis, J. M. Cushing, and J. Hyman, "Bifurcation analysis of a mathematical model for malaria transmission," *SIAM Journal on Applied Mathematics*, vol. 67, no. 1, pp. 24–45, 2006.

[2] N. Chitnis, J. M. Hyman, and C. A. Manore, "Modelling vertical transmission in vector-borne diseases with applications to rift valley fever," *Journal of biological dynamics*, vol. 7, no. 1, pp. 11–40, 2013.

[3] M. J. Keeling and P. Rohani, *Modeling Infectious Diseases in Humans and Animals*, vol. 47. 2007.

[4] C. P. Farrington, M. N. Kanaan, and N. J. Gay, "Branching process models for surveillance of infectious diseases controlled by mass vaccination.," *Biostatistics (Oxford, England)*, vol. 4, no. 2, pp. 279–295, 2003.

[5] A. L. Lloyd, J. Zhang, and A. M. Root, "Stochasticity and heterogeneity in host-vector models.," *Journal of the Royal Society, Interface / the Royal Society*, vol. 4, no. 16, pp. 851–863, 2007.

[6] P. Jagers, "Branching processes with biological applications," *New York: Wiley*, 1975.

[7] S. Karlin and H. Taylor, "A first course in stochastic processes," *New York: Academic Press*, vol. 2nd ed., 1975.

[8] G. E. Lahodny and L. J. S. Allen, "Probability of a Disease Outbreak in Stochastic Multipatch Epidemic Models," *Bulletin of Mathematical Biology*, vol. 75, no. 7, pp. 1157–1180, 2013.

[9] G. Grimmett and D. Stirzaker, "Probability and random processes," *Oxford, UK: Oxford University Press*, 1992.

[10] K. Athreya and P. Ney, "Branching processes," *Berlin, Germany: Springer.*, 1972.

[11] N. Van Kampen, "Stochastic Processes in Physics and Chemistry," 2007.

[12] A. McKane and T. Newman, "Stochastic models in population biology and their deterministic analogs," 2004.

[13] R. H. Wang, Z. Jin, Q. X. Liu, J. van de Koppel, and D. Alonso, "A simple stochastic model with environmental transmission explains multi-year periodicity in outbreaks of avian flu," *PLoS ONE*, vol. 7, no. 2, 2012.

[14] D. Alonso, A. J. McKane, and M. Pascual, "Stochastic amplification in epidemics.," *Journal of the Royal Society, Interface / the Royal Society*, vol. 4, no. 14, pp. 575–582, 2007.
